# The European Ruminants during the “*Microbunodon* Event” (MP28, Latest Oligocene): Impact of Climate Changes and Faunal Event on the Ruminant Evolution

**DOI:** 10.1371/journal.pone.0116830

**Published:** 2015-02-18

**Authors:** Bastien Mennecart

**Affiliations:** 1 CR2P - Centre de Recherches sur la Paléobiodiversité et les Paléoenvironnements, UMR 7207, Muséum National d’Histoire Naturelle, CNRS, UPMC, Sorbonne Universités. MNHN, CP38, 8 rue Buffon, 75005 Paris, France; 2 SNMB-Bayerische Staatssammlung für Paläontologie und Geologie, Munich, Germany; New York Institute of Technology College of Osteopathic Medicine, UNITED STATES

## Abstract

The Earth already experienced numerous episodes of global warming and cooling. One of the latest impressive events of temperature rising was the Late Oligocene Warming that occurred around 25 Mya. An increase of the marine temperature of 2 to 4°C has been observed in a short time interval. In Europe, this major climatic event can be correlated to the continental faunal turnover “*Microbunodon* Event”. This event is marked by a huge faunal turnover (40% of the ungulate fauna during the first 500k years) and environmental changes. Drier conditions associated to the appearance of the seasonality lead to new environmental conditions dominated by wooded savannahs. This is correlated to a major arrival of Asiatic immigrants. Moreover, from a homogenous fauna during the main part of the Oligocene, local climatic variations between the European Western coast and the more central Europe could have provided faunal regionalism during the latest Oligocene and earliest Miocene. Considering the ruminants, this event is the major ever known for this group in Europe. A total renewal at the family level occurred. Thanks to a precise stratigraphic succession, major evolutionary elements are highlighted. Typical Oligocene species, mainly Tragulina, were adapted to wooded environments and were leaves/fruits eaters. They disappeared at the end of MP27 or the early MP28. This corresponds to the appearance of the Asiatic immigrants. The Tragulina (Lophiomerycidae, Bachitheriidae) and stem Pecora gave way to more derived stem and maybe crown Pecora (e.g. “*Amphitragulus*”, *Babameryx*, *Dremotherium*). These newcomers were adapted to more open environments and mixed feeding. The disappearance of the Tragulina is probably linked to environmental and vegetation changes, and competition. They give way to more derived ruminants having a more efficient metabolism in drier conditions and a better assimilation of less energetic food.

## Introduction

Nowadays, the comprehension of the current global warming event is a major asset to prevent extinctions [1]. As consequences of the temperature rise, environments and disease dynamics are changing leading to populations’ migrations and widespread extinctions [2], [3] Down to the past, major climate changes already occurred giving information on such events. During the Oligocene, two major climatic events conducted to important faunal renewal in Europe.

The “Grande Coupure” Event marks the base of the Oligocene [4]. This already well-known event is directly linked to the climatic and tectonic Terminal Eocene Event [4]–[6]. During the Eocene, Europe was mainly isolated from the other continents. The cooling event from the Terminal Eocene Event (a decrease of around 6°C recorded in Northern Atlantic organisms, [6]) concluded to the formation of a permanent ice sheet in Antarctica [6] and the dry out of the Turgai straits in Asia [5]. It permitted the immigration of Asiatic animals into Europe and the extinction of European endemic fauna with a faunal renewal of 60% [4–7]. The “*Microbunodon* Event” also shows a major faunal renewal (40% of the ungulates) during a very short time interval (MP28, between 24.8Mya and 24Mya; see [8], [9]). It seems to be linked to the global climatic event, described in marine sediments, named “Late Oligocene Warming” [6]. This continental faunal event has been named after the emblematic anthracotheriid artiodactyl, which immigrated from Asia during MP28 [9]. A rise of the temperature of 2 to 4°C is observed in marine organisms from North Sea and Northern Atlantic [10], [11]. This faunal turnover is observed among the entire group of vertebrates and plants (terrestrial plants [12]; charophyta [13]; booid snakes [14]; micromammals [15]; and large mammals, especially the ruminant, which the fauna is totally renewed [8], [9], [16]–[19]). This event is still not well-understood.

The replacement modalities in the composition of the ruminant fauna during the “*Microbunodon* Event” remain unclear. Currently, the record of the transition is mainly based on the Pech Desse (early MP28) and Pech du Fraysse (MP28 type locality) localities from the Quercy [17]. Blondel [17] already noticed that the ruminant fauna changed drastically and rapidly during the MP28 time range. The oldest locality Pech Desse (South-western France) yielded the primitive Oligocene taxa *Lophiomeryx*, *Bachitherium*, and *Prodremotherium*, when they are all already absent in Pech du Fraysse (South-western France). Moreover, Pech Desse is the only dated locality where *Prodremotherium elongatum* and *Bachitherium lavocati* are currently described [17], [20], [21].

**Fig 1:**
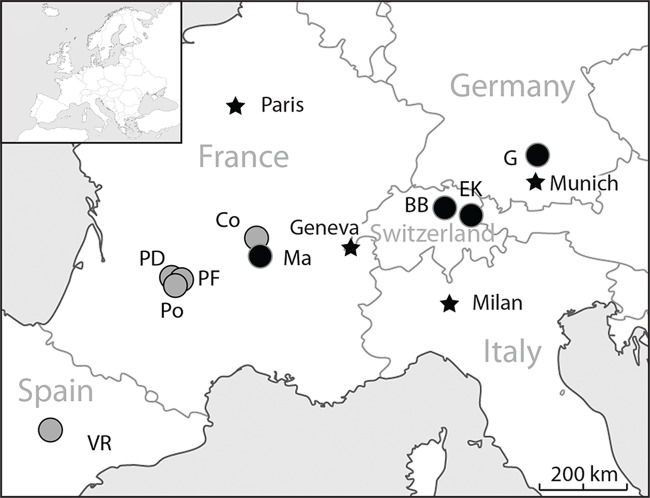
Location of the main MP28 ruminant localities in Western Europe. BB Balstahl Bännli; Co Cournon; EK Ebnat Kapel; G Gaimersheim1; Ma Mailhat; PD Pech Desse; PF Pech du Fraysse; Po Portal; VR Vivel del Rio. Grey dots data from the literature; Black dots new data.

The primary aim of this article is to describe ruminant taxa present in European localities dated to MP28 in order to better know the anatomy and variability of these mammals and clarify their biostratigraphy. The reassessment of the ruminant fossils from long time known localities (the most important in number of fossils and/or diversity being: Gaimersheim1, Germany, MP28, [22]; and Mailhat, France, undefined Oligocene between MP23 and MP28, [23], Fig. 1) gives access to new unsuspected data on the ruminant evolution. Then, a discussion of the faunal changing through their synecological, the climate and environmental changes, highlights the factors that may be involved in this major faunal turnover.

## Historical & Geological Background of the Localities

This study presents first or new descriptions of fossil material from Western Europe localities, dated to MP28. Mailhat locality, also written “Malhat” and “Croix de Saint-Vernet” [23], [24], is an old known French paleontological locality. Bravard first described in “Croix-de-Saint-Vernet, Western Malhat“, bone fragments attributed to the Perissodactyla *Lophiodon* [25]. However, after reevaluation of this material, he concluded to the presence of “*Rhinoceros*” *medius*, an attribution that he recognized as doubtful [23], [24], [25], [26]. This species does not seem valid anymore and no reassessment of the material has been performed. Following Giraud [26] and Bravard [24], there is an *Anthracotherium cyclognatum* in “Vernet, Western Malhat”. Hugueney [23] synonymised it with *Anthracotherium magnum* without any explanations. Nevertheless, a doubt still persists on the presence of this animal in this locality, because *A*. *magnum* has been found in other localities between Orsonnette and Lamontgie, close to Mailhat. A complete mandible of *Anthracotherium magnum* [27] has been recognized by the previous authors [24], [25]. A mandible of *Cainotherium*?, similar to those in Phosphorites du Quercy and smaller than those of Auvergne (Saint-Gérand le Puy, Montaigu le Blin) is also described in Malhat, près Lamontgie [26]. The ruminant remains were first all attributed to *Cervus malhaticus* nov. sp. by Mr. Bravard (manuscript). The reassessment of these fossils by Lavocat [28] concluded that these remains (Br.12, two upper dentition rows) belong to *Lophiomeryx chalaniati*. This determination is followed by Brunet & Sudre [29] and Hugueney [23]. However, the size and shape of the specimens are too different to belong to only one species. Additional ruminant specimens from this locality have been observed. Unfortunately, no micromammals had been described for Mailhat. Thus, the current macromammals faunal list proposed by Hugueney [23] is: *Anthracotherium magnum* (*Anthracotherium cyclognatum* of Bravard [24] that necessitate reassessment), *Rhinoceros medius*? (first described as *Lophiodon* and requiring a reassessment), *Cainotherium* sp., and *Lophiomeryx chalaniati*. Hugueney [23] proposes an approximate age comprise between MP23 and MP28 [23].

Dated to MP28 [22], the German locality of Gaimersheim1 is known since the first half of the 19^e^ century. The abundance and diversity of the fossils found in this fissure filling made numerous authors able to study its herpetofauna [30–32], its avifauna [33], and its mammals (micromammals [34–37]; carnivores [38]; ungulates: [39] for the tapirids, [40] for the cainotheriids, and [41] for the rhinocerothids). However, besides Geraads *et al*. [42], who mentioned the presence of an advanced *Bachitherium* with fused metacarpals from Gaimersheim, up to now, no further information on the ruminant material has been provided.

Ruminants from two Swiss localities already mentioned in Mennecart 2012 [8], have been integrated in this study (Fig. 1). Balstahl Bännli belongs to the Knauermolasse Formation (synonym of the Aarwangen Molasse and lateral equivalent of the Molasse Alsacienne Formation within the eastern Jura Molasse and the distal Plateau Molasse; [43–45]). Following the preliminary analysis of the small mammals (manuscript note in the collection of the Natural History Museum of Basel), this locality has been dated to MP28.

Ebnatt Kappel belongs to the Ebnater Schichten (Untere Süsswasser Molasse). The micromammals of this locality (Theridomyidae, Cricetidae and Eomyidae) are closely related to those of Gaimersheim and Pech du Fraysse [46]. Ebnatt Kappel is dated to MP28 [9], [47]. No less than 26 plant species (see [48] for a complete list), and four large mammals (*Protopirus bavaricus*, *Ronzotherium romani*, *Microbunodon minimum*, *Dremotherium* cf. *quercyi* [49]) are described.

## Material & Methods

### Material

The referred material is stored in the Bayerische Staatssammlung für Paläontologie und Geologie (München, Germany: Gaimersheim1), the Muséum National d’Histoire Naturelle (Paris, France: Mailhat), the Université Montpellier 2 (France: Pech Desse and Pech du Fraysse), the Naturmuseum Solothurn (Switzerland: Balstahl Bännli), and the private collection of Urs Oberli (St Gallen, Switzerland: Ebnat Kappel). Already mentioned by Scherler *et al*. [47], the latter is a “non-profit institution” and is accessible to the public. “Visits can be arranged with the Naturhistorisches Museum of St. Gallen”. No permits were required for the described study, which complied with all relevant regulations. The acronyms used are **SNSB-BSPG** Bayerische Staatssammlung für Paläontologie und Geologie (München, Germany); **MNHN** Muséum National d’Histoire Naturelle (Paris, France); **NMO** Naturmuseum Olten (Switzerland), **NMS** Naturmuseum Solothurn (Switzerland); **USTL** Montpellier 2 (France).

The studied ruminant material only belongs to localities dated to MP28, as this time period corresponds to one of the main faunal turnover with the “Grande Coupure” during the Paleogene. Even if all described ruminants species are already known by several publications (e.g. [17], [20], [50], [51]), new anatomical features are presented here (mandible, teeth and postcranial bones). *Prodremotherium elongatum* specimens have been the topic of a recent article [52]. Thus they have not been redescribed and reassessed in this work. The main part of the present specimens is unpublished. The ruminants from Gaimersheim1 are for the first time labelled and described. The ruminants from Mailhat were only known by two specimens numbered Br.12 (two upper dentitions: P4-M2 and M2-M3). Unfortunately, only one fossil has been found. Its reinterpretation leads to the description of the tooth row D4-M2 of *Lophiomeryx chalaniati*. The other ruminant specimens from Mailhat were unpublished. The Swiss specimens were already mentioned in previous works [6], [49], but not firmly described. The ruminants from Pech Desse and Pech du Fraysse have been observed and described, when new characteristics (mandible shape for example) were not included in Blondel [17]. This article presents for the first time the pictures and additional unpublished views of the holotypes of *Bachitherium lavocati* and “*Amphitragulus*” *quercyi*.

### Methods

**Fig 2:**
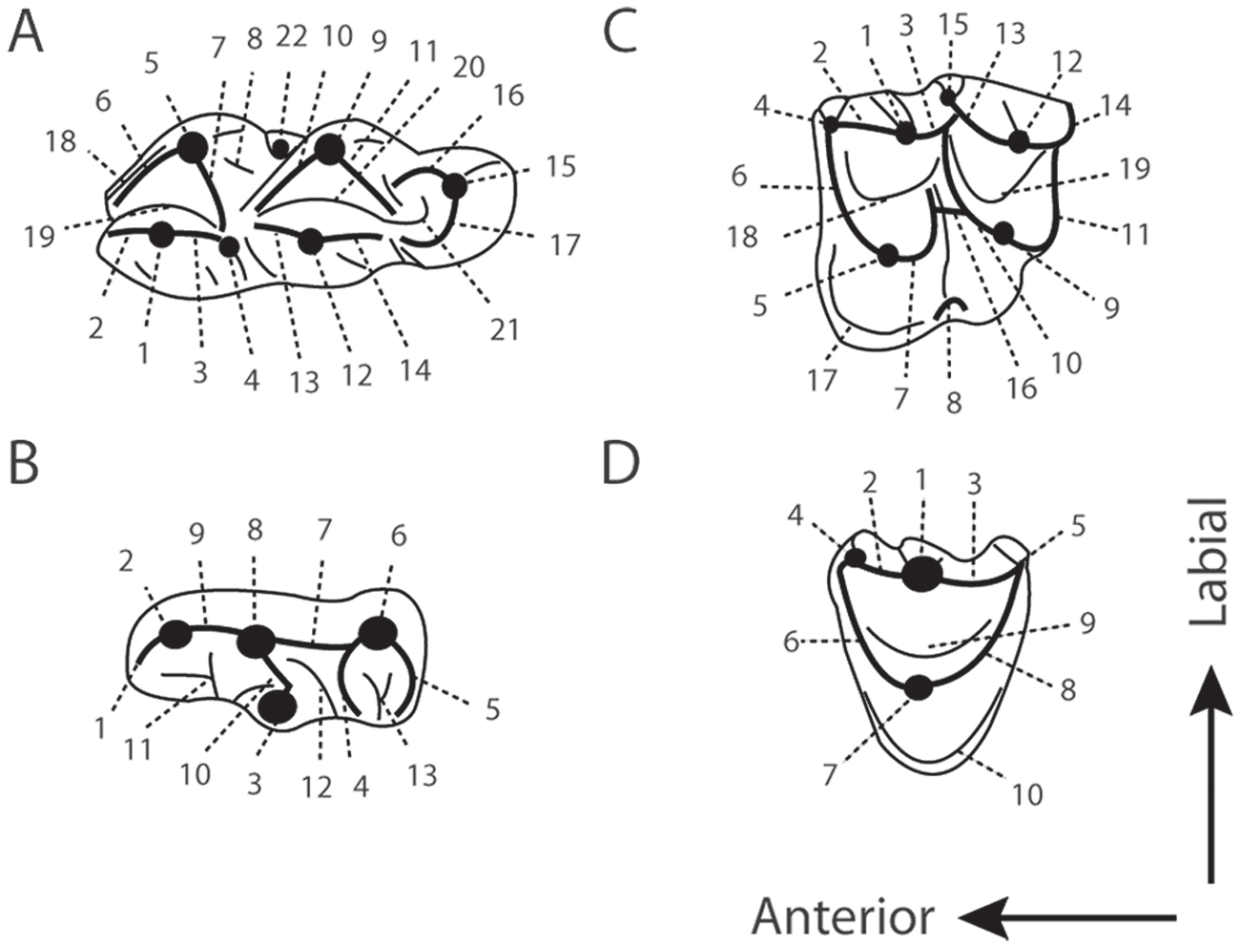
Ruminant dental terminology. **A**, Lower molars. 1, metaconid; 2, premetacristid; 3, postmetacristid; 4, metastylid; 5, protoconid; 6, preprotocristid; 7, internal postprotocristid; 8, external postprotocristid; 9, hypoconid; 10, prehypocristid; 11, posthypocristid; 12 entoconid; 13, preentocristid; 14, postentocristid; 15 hypoconulid; 16, prehypoconulidcristid; 17, posthypoconulidcristid; 18, anterior cingulid; 19, anterior basin; 20, posterior basin; 21, back basin of m3; 22, ectostylid. **B**, Lower premolars. 1, anterior stylid; 2, anterior conid; 3, mesolingual conid; 4, posterolingual conid; 5, posterior stylid; 6, posterolabial conid; 7, posterolabial cristid; 8, mesolabial conid; 9, anterolabial cristid; 10, transverse cristid; 11, anterior valley; 12, posterior valley; 13, back valley. **C**, Upper molars. 1, paracone; 2, preparacrista; 3, postparacrista; 4, parastyle; 5, protocone; 6, preprotocrista; 7, postprotocrista; 8, entostyle; 9, metaconule; 10, premetaconulecrista; 11, postmetaconulecrista; 12, metacone; 13, premetacrista; 14, postmetacrista; 15, mesostyle; 16, premetaconulecrista bifurcation; 17, lingual cingulum; 18, anterior fossa; 19, posterior fossa. **D**, Upper premolars. 1, anterolabial cone; 2, anterolabial crista; 3, posterolabial crista; 4, anterior style; 5, posterior style; 6, anterolingual crista; 7, lingual cone; 8, posterolingual crista; 9, fossa; 10, lingual cingulum. Modified from Bärmann & Rössner [53] and Mennecart & Métais [52].

The dental terminology follows Bärmann & Rössner [53] (see Fig. 2) and that of the mandible Mennecart *et al*. [54]. First, second, and third lower molars are designated as m1, m2, and m3, respectively, and first, second, and third upper molars as M1, M2, and M3. Similarly, upper and lower permanent premolars are respectively designated as p1, p2, p3, p4, and P2, P3, P4, and upper and lower decidual premolars as d1, d2, d3, d4, and D2, D3, D4. The lower canine is named as c and the metatarsal bones as Mt. The astragalus morphology and measurements follow Martinez & Sudre [21]. Uncertain generic attribution for “*Amphitragulus*”*quercyi* is expressed with quotation marks. Cranial material of *Bachitherium lavocati* and “*Amphitragulus*” *quercyi* is not described in detail in this article and will be the subject of a separate study. Sizes are given in mm with a precision of 0.2 mm. The first data is the length; the second one is the width.

The chronostratigraphical framework is based on geological time scales for the Paleogene [4], [55]. Classically, European Land Mammal Ages (ELMA) have been described has a succession of reference levels defined by type localities. These localities have been chosen according to their mammal diversity and their geographical location [56]. This relative dating method was first applied to avoid the problem of diachronism among boundaries of various area [56]. Moreover, it was not possible to associate dating that you need using biozones, because “*the resolution power of absolute dating* was *too weak given the fact that much too few calibrations* were *available for the European Paleogene and their inaccuracy* was *of the same order of magnitude as the intervals to be measured*” [56]. However, Hooker noted that “*In practice*, *because of referral of a fauna other than the reference fauna to a given reference level*, *a temporal range is spanned and a reference level is thus used in much the same way as a standard assemblage biozone*” [4]. Moreover, new data, based on magnetostratigraphy and ^40^Ar/^39^Ar radiometric dating, improve the dating of these Paleogene biozones [57], [58]. Thus, numerous authors use biozones instead of reference levels in the Paleogene of Europe (e.g. [23], [47], [59]). To facilitate the reading and the comprehension of this article, biozones will be used instead of reference levels. Successions and durations of Oligocene mammal zones are established after Vandenberghe *et al*. [4]. The chosen time period encompasses the faunal transition occurring during the “*Microbunodon* Event “. To highlight the significance of the ruminant faunal changes during this period, comparisons with older and younger localities is done thanks to recently published data. The poly-cohort analysis follows Scherler *et al*. [9]. However, contrary to Scherler *et al*. [9], a focus on ruminant at the species level is done, integrating the new data presented here.

## Systematic Paleontology

*Class **MAMMALIA** Linnaeus, 1758*

*Order **CETARTIODACTYLA** Montgelard, Catzeflis, and Douzery, 1997*

*Suborder **RUMINANTIA** Scopoli, 1777*

*Family **LOPHIOMERYCIDAE** Janis, 1987*

*Genus **Lophiomeryx** Pomel, 1853*

***Lophiomeryx chalaniati** Pomel, 1853*

Fig. 3

**Fig 3:**
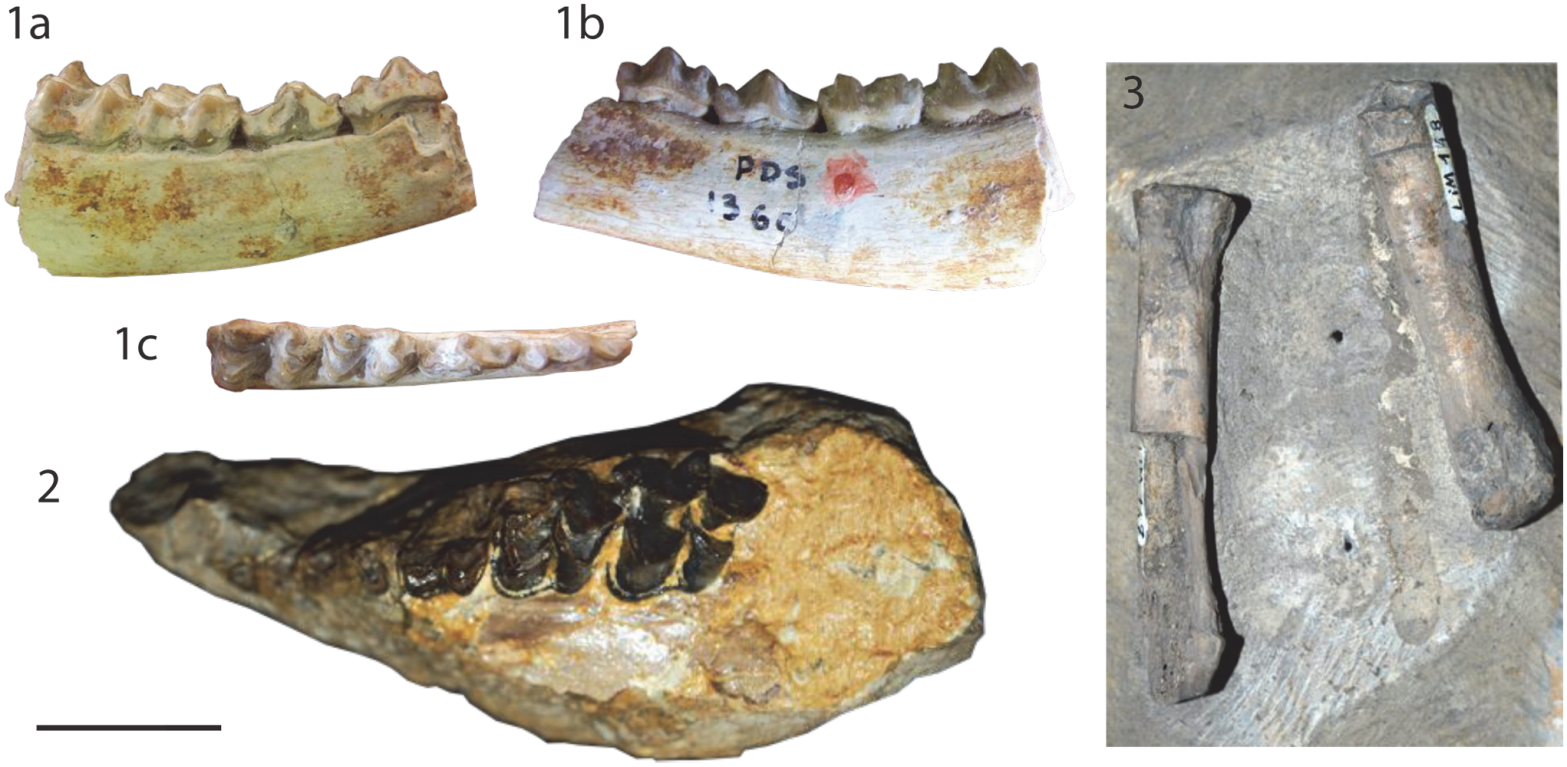
Specimens of *Lophiomeryx chalaniati*. from *Pech Desse*: **1** right mandible with the tooth row p3-m2 (USTL PDS1360), (a) labial, (b) lingual, and (c) occlusal views. from *Mailhat* **: 2** left maxillary with roots of D2 and D3, broken D4, and M1-M2 (MNHN LIM150); **3** right metatarsal II and partial bone III (MNHN LIM148). The scale bar is 2 cm.

### Holotype & referred specimens information

**Holotype.** Following Brunet & Sudre [29], we consider as holotype for the species *Lophiomeryx chalaniati* the specimen BM34960, a left mandible with d3-m3 ([60], Fig. 17; [29], Fig. 16), from La Sauvetat (MP25, France).

**Referred specimens.** *Mailhat* **MNHN LIM148** right metatarsal II and fragment of metatarsal III (mid distal part) (this work, Fig. 3.3); **MNHN LIM150** juvenile left maxillary with D4–2 (M3 is not erupted), and the tooth sockets of D3 and D2 (old number in [28] Br.12 Cerv. Malh.; this work, Fig. 3.2). *Pech Desse* **USTL PDS1360** right corpus mandibulae with m2–3 ([8], p.81, Fig. 15; this work, Fig. 3.1).

**Measurements.** MNHN LIM148 MtII (L 54.8; proximal. width 10.7), MtIII (distal width 10.5); MNHN LIM150 M1 (?x14?) M2 (15.5x16.2).

### Description

**Upper deciduous dentition.** All crowns are broken. However, we can observe that D2 is biradiculate and D3 possesses three roots. The D3 is very anteroposteriorly elongated (~12x7mm) with a metaconule relatively salient with a rounded base. The D4 is well-molarized being square in occlusal view. The labial cusps are missing. The protocone is enlarged having the postprotocrista posteriorly oriented and reaching the premetaconulecrista at its middle. The metaconule is narrow and v-shaped in occlusal view with a straight premetaconulecrista. The lingual cingulum is strong and surrounds the entire protocone.

**Upper permanent dentition.** The upper molars have relatively square outline M2 being larger than M1. The cusps are very selenomorph. The fossae have flat lingual surfaces. The protocone is widely distally open and larger than the metaconule. The preprotocrista is straight and labially oriented reaching the prominent but broken parastyle. The postprotocrista is oblique and joins the premetaconulecrista at its middle part, at a slightly lower height. The premetaconulecrista is elongated, thin, straight, and oblique. It finishes at the level of the mesostyle, located side by side to the labial wall. The postmetaconulecrista is slightly concave and joins the postmetacrista. The metaconule is V-shaped in occlusal view. The paracone and the metacone are aligned labially. The weak paracone labial rib is stronger than that of the labially concave metacone, whereas both possess relatively short and straight cristae. The mesostyle and the parastyle, even if broken, are strong, salient and off-centred from the axis of the labial part of the paracone and the metacone. A metastyle is absent. The anterior cingulum is very strong surrounding the protocone. There is a weaker posterior cingulum, which stops at the end of the metaconule.

**Metatarsals.** The metatarsal II and III are partly broken. Due to preparation, neither the anterior view can be studied on the MtII nor the plantar view of the MtIII. Both are straight and keep the same size all along the diaphysis. Both bones are fully separated and with a similar thickness of the diaphysis. The better preserved (MtII) has the plantar part broken. The proximal articulation is triangular in shape, with a small depression on its plantar view. A distal rugosity is well marked. The proximal half of the MtIII is missing and the distal keel is worn.

### Discussion

MNHN LIM150 possesses an association of primitive characteristics relatively unique among the European ruminants, which can easily exclude it from several attributions. Numerous ruminants of various families all along the Oligocene possess a lingual cingulum on upper molars: *Lophiomeryx*, *Iberomeryx*, *Gelocus*, *Mosaicomeryx*, *Babameryx* [8], [42], [50]–[52], [54], [60]–[63]. The selenodont teeth (associated with an enlarged size) are never observed in *Iberomeryx* and *Gelocus* [42], [54], [52]. *Mosaicomeryx* and *Babameryx* are stem Pecora, which possess a long and labially oriented postprotocrista [52], [62]. Moreover, the styles in MNHN LIM150 are very enlarged, which is not the case of *Mosaicomeryx* and *Babameryx*. The teeth description of MNHN LIM150 corresponds to those of *Lophiomeryx* [29], [51], [63]. Moreover, in all ruminants from the European Oligocene, the metatarsals II and III are fused except in *Lophiomeryx* [42], [54], [52], [61]. The differentiation of European *Lophiomeryx* species is mainly based on size differences [29], even if Antoine *et al*. [51] suggests that *Lophiomeryx mouchelini* may possess specimens as large as *Lophiomeryx chalaniati* representatives during the late Suevian. MNHN LIM150 teeth are clearly in the population variability of *Lophiomeryx chalaniati* observed by Filhol [64], Brunet & Sudre [29], and Mennecart [8], and larger than *Lophiomeryx mouchelini* populations [8], [29]. *L*. *chalaniati* is the only known European *Lophiomeryx* during the entire Arvenian (mostly Late Oligocene).

The specimen MNHN LIM148 is very interesting in completing the description of the central metacarpals attributed to a European *Lophiomeryx*, made by Brunet & Sudre ([29], USTL ACQ 328 from the old collection of Quercy) and by Schlosser ([65], Pl.2, Figs 13 & 14). MNHN LIM148 differs from the Early Oligocene from Mongolia *Lophiomeryx gobiae* described by Trofimov [66] in having shorter external metapodials II and V. However, Métais & Vislobokova [63] do not consider that *Lophiomeryx gobiae* belong to the genus *Lophiomeryx* and Vislobokova [67] proposed that this species is a Hypertragulidae. Nevertheless, the reduction of the lateral digits into bonny stylus appeared several times during the evolution of the ruminants [68].

*Family **BACHITHERIIDAE** Janis, 1987*

*Genus **Bachitherium** Pomel, 1853*

***Bachitherium lavocati** Sudre, 1986*

Fig. 4

**Fig 4:**
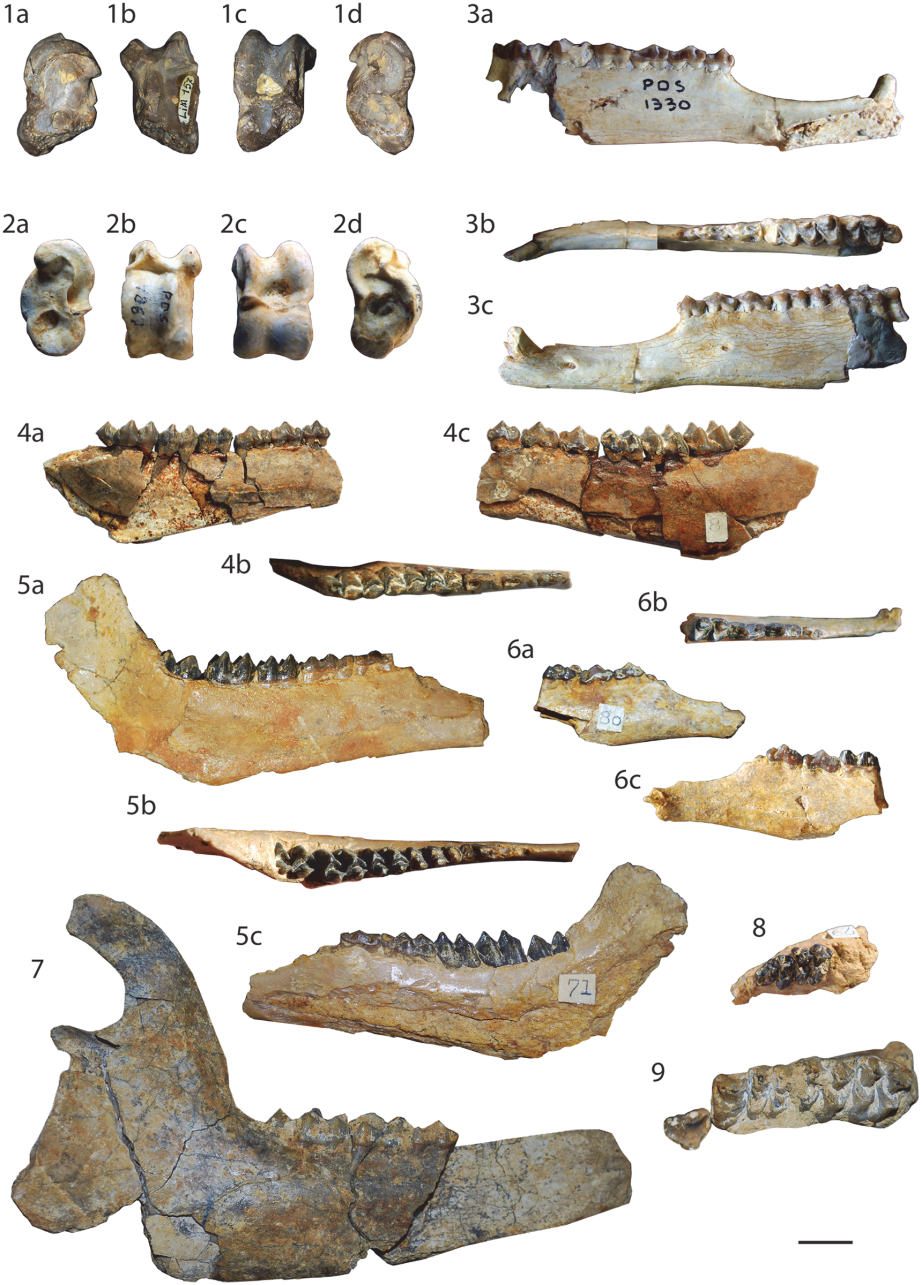
Specimens of *Bachitherium lavocati* and *Bachitherium* cf. *lavocati*. *Bachitherium* cf. *lavocati* from *Mailhat*: **1** right astragalus (MNHN LIM157), (a) medial, (b) plantar, (c) anterior, and (d) lateral views. *Bachitherium lavocati* from *Pech Desse*: **2** left astragalus (USTL PDS1863), (a) medial, (b) plantar, (c) anterior, and (d) lateral views; **3** mandible with the teeth p1-m3 (USTL PDS1330, holotype), (a) lingual, (b) occlusal, and (c) labial views. *Bachitherium lavocati* from *Gaimersheim1*: **4** left corpus with the tooth row p2-m3 (SNSB-BSPG 1952II4953), (a) lingual, (b) occlusal, and (c) labial views; **5** left mandible with the tooth row d3-m3 (SNSB-BSPG 1952II4833), (a) lingual, (b) occlusal, and (c) labial views; **6** right partial corpus with p3-m1 (SNSB-BSPG 1952II4826), (a) labial, (b) occlusal, and (c) lingual views; **7** right mandible with m1-m3 (SNSB-BSPG 1952II4910); **8** left maxillary fragment with D3-D4 (SNSB-BSPG 1952II4857); **9** left maxillary fragment with the tooth row P3-M3 (SNSB-BSPG 1952II4902). The scale bar is 1 cm.

### Holotype & referred specimens information

**Fig 5:**
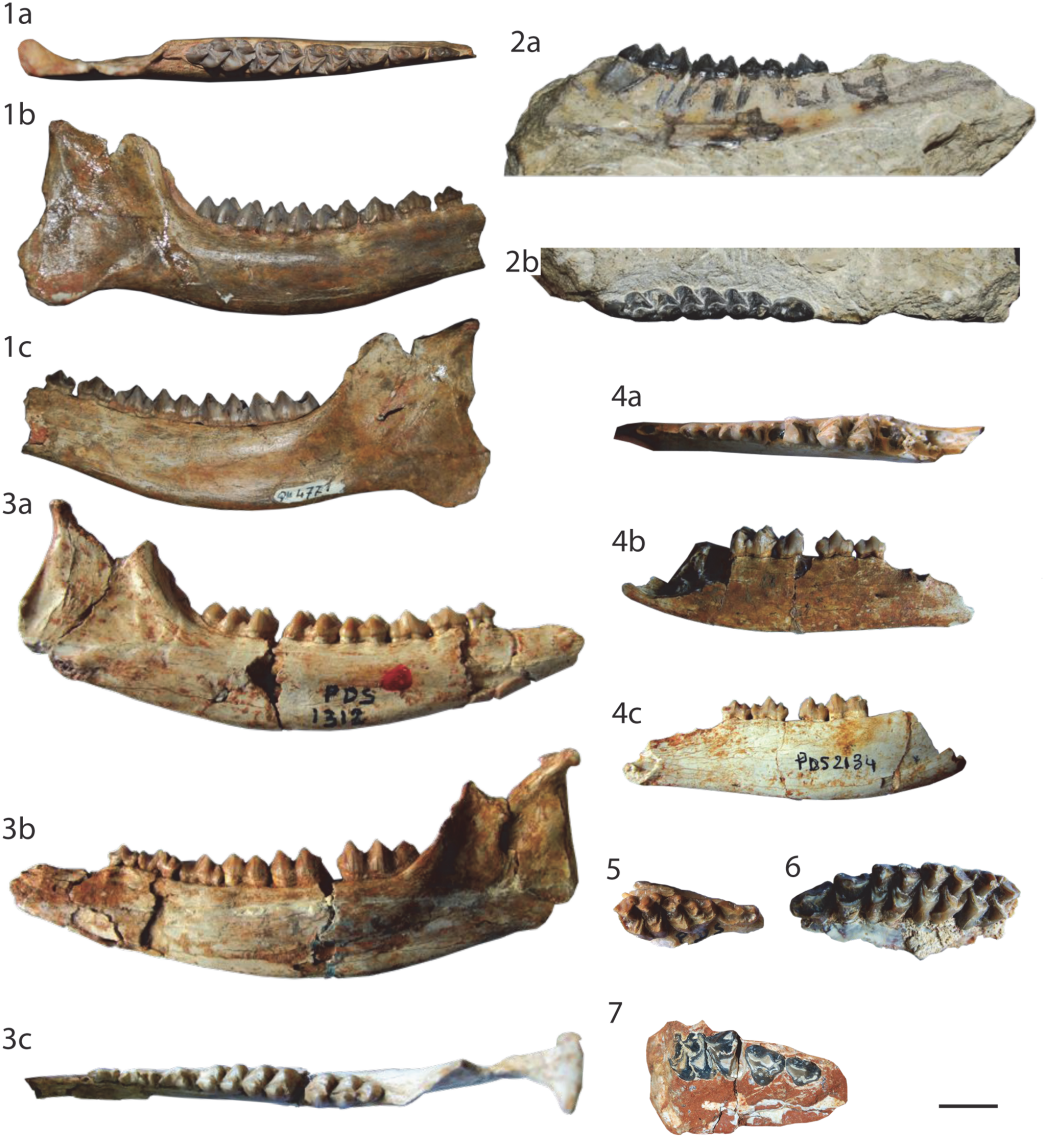
Specimens of *“Amphitragulus” quercyi*. from *Old Quercy collections*: **1** right mandible with p2-m3 (MNHN Qu4771, holotype), (a) occlusal, (b) labial, and (c) lingual views. from *Balsthal Bännli*: **2** left mandible with p4-m3 (NMS 7685), (a) lingual and (b) occlusal views. from *Pech Desse*: **3** right mandible with the tooth socket of p1 and p2-m3 (USTL PDS1312), (a) labial, (b) lingual, and (c) occlusal views; **4** right mandible with the tooth socket of p1 and the tooth row p2-m1 (USTL PDS2134),), (a) occlusal, (b) labial, and (c) lingual views; **5** right maxillary fragment with D2-D4 (USTL PDS1421); **6** left maxillary fragment with P3-M3 (USTL PDS2139). from *Gaimersheim1*: **7** right maxillary fragment with P2-M1 (SNSB-BSPG 1952II4912). The scale bar is 1 cm.

**Holotype.** The holotype of *Bachitherium lavocati*, established by Sudre [20], is a left mandible with the root of p1 and p2-m3, USTL PDS1330 ([17], Fig. 1.1; [20], Fig. 5; this work, Fig. 4.3), from Pech Desse (MP28, France).

**Referred specimens.** *Pech Desse* **USTL PDS1863** left astragalus (this work, Fig. 4.2); **USTL PDS1330** left mandible with the teeth p1-m3 (Holotype; [17], Fig. 1.1; [20], Figs 4 & 5; this work, Fig. 4.3). *Gaimersheim1* **SNSB-BSPG 1952II4800** right mandible with p2-p3; **SNSB-BSPG 1952II4801** right m1; **SNSB-BSPG 1952II4802** left mandible with p4-m3; **SNSB-BSPG 1952II4805** left maxillary with D3-D4; **SNSB-BSPG 1952II4806** left maxillary with D3-D4; **SNSB-BSPG 1952II4807** left D3; **SNSB-BSPG 1952II4808** left D3; **SNSB-BSPG 1952II4809** right D3; **SNSB-BSPG 1952II4810** right p3; **SNSB-BSPG 1952II4812** right mandible with m2-m3; **SNSB-BSPG 1952II4813** right mandible with p3-p4; **SNSB-BSPG 1952II4814** left mandible with p2; **SNSB-BSPG 1952II4815** left mandible with m1-m3; **SNSB-BSPG 1952II4816** right mandible with m3; **SNSB-BSPG 1952II4817** right mandible with m1-m3; **SNSB-BSPG 1952II4819** left mandible with m2-m3; **SNSB-BSPG 1952II4822** left mandible with m1-m2; **SNSB-BSPG 1952II4823** right mandible with p3-p4; **SNSB-BSPG 1952II4824** left mandible with p3-m1; **SNSB-BSPG 1952II4825** right mandible with m2-m3; **SNSB-BSPG 1952II4826** right mandible with p3-m1 (this work, Fig. 4.6); **SNSB-BSPG 1952II4827** left P3 and P4; **SNSB-BSPG 1952II4828** right d4; **SNSB-BSPG 1952II4829** right mandible with d4; **SNSB-BSPG 1952II4830** left mandible with d4; **SNSB-BSPG 1952II4831** left M1; **SNSB-BSPG 1952II4832** left M1; **SNSB-BSPG 1952II4833** left mandible with d3-m3 (this work, Fig. 4.5); **SNSB-BSPG 1952II4834** left mandible with d4-m2; **SNSB-BSPG 1952II4835** right m1; **SNSB-BSPG 1952II4836** right M2; **SNSB-BSPG 1952II4837** left mandible with m3; **SNSB-BSPG 1952II4838** diastema; **SNSB-BSPG 1952II4839** right mandible with p4-m1; **SNSB-BSPG 1952II4840** left mandible with p3-m1; **SNSB-BSPG 1952II4841** right mandible with d3-m1; **SNSB-BSPG 1952II4842** right mandible with m1-m3; **SNSB-BSPG 1952II4843** left m2; **SNSB-BSPG 1952II4846** left mandible with m1-m2; **SNSB-BSPG 1952II4848** left mandible with m2-m3; **SNSB-BSPG 1952II4849** right mandible with m1-m3; **SNSB-BSPG 1952II4850** right and left mandibles with both m3; **SNSB-BSPG 1952II4852** left mandible with p2-m3; **SNSB-BSPG 1952II4853** left mandible with m3; **SNSB-BSPG 1952II4856** left maxillary with M1-M3; **SNSB-BSPG 1952II4857** left maxillary with D3-D4 (this work, Fig. 4.8); **SNSB-BSPG 1952II4859** right M1; **SNSB-BSPG 1952II4860** right M2; **SNSB-BSPG 1952II4861** left m2; **SNSB-BSPG 1952II4862** right mandible with d2-d4; **SNSB-BSPG 1952II4863** left mandible with d3; **SNSB-BSPG 1952II4864** right d3; **SNSB-BSPG 1952II4866** right m3; **SNSB-BSPG 1952II4869** left P2; **SNSB-BSPG 1952II4869** left mandible with m1 & p4-m1; **SNSB-BSPG 1952II4871** left m2; **SNSB-BSPG 1952II4874** right M3; **SNSB-BSPG 1952II4875** right m2; **SNSB-BSPG 1952II4876** right m3; **SNSB-BSPG 1952II4877** left m3; **SNSB-BSPG 1952II4879** right mandible with m2-m3; **SNSB-BSPG 1952II4880** right p2; **SNSB-BSPG 1952II4881** right mandibles with p4-m1, right m2 and left p2-p4; **SNSB-BSPG 1952II4882** left P3; **SNSB-BSPG 1952II4883** right mandible with p3-m1 and left M1; **SNSB-BSPG 1952II4886** mandible with m1 right; **SNSB-BSPG 1952II4887** right p3; **SNSB-BSPG 1952II4888** right m; SNSB-BSPG **1952II4889** left d4; **SNSB-BSPG 1952II4890** right d3; **SNSB-BSPG 1952II4893** left mandible with p2-m3; **SNSB-BSPG 1952II4895** left mandible with d4-m3; **SNSB-BSPG 1952II4897** fragmented skull; **SNSB-BSPG 1952II4898** fragmented skull; **SNSB-BSPG 1952II4899** fragmented skull; **SNSB-BSPG 1952II4900** fragmented skull; **SNSB-BSPG 1952II4901** fragmented skull; **SNSB-BSPG 1952II4902** fragmented skull (this work, Fig. 4.9); **SNSB-BSPG 1952II4903** fragmented skull; **SNSB-BSPG 1952II4904** fragmented skull; **SNSB-BSPG 1952II4905** left mandible with m1-m3; **SNSB-BSPG 1952II4906** left mandible with p3-p4; **SNSB-BSPG 1952II4907** right mandible with p3 m1-m2; **SNSB-BSPG 1952II4908** right mandible with p2-m3; **SNSB-BSPG 1952II4910** right mandible with m1-m3 (this work, Fig. 4.7); **SNSB-BSPG 1952II4913** mandible with p3-m3; **SNSB-BSPG 1952II4916** right maxillary with D3-M1; **SNSB-BSPG 1952II4918** right maxillary with P3-M1; **SNSB-BSPG 1952II4919** right M3; **SNSB-BSPG 1952II4922** left maxillary with P4-M3; **SNSB-BSPG 1952II4923** left maxillary with P4-M3; **SNSB-BSPG 1952II4925** left maxillary with M1-M3; **SNSB-BSPG 1952II4926** left maxillary with M1-M3; **SNSB-BSPG 1952II4928** right maxillary with M1-M2; **SNSB-BSPG 1952II4929** left M3; **SNSB-BSPG 1952II4930** left M1; **SNSB-BSPG 1952II4931** right M3; **SNSB-BSPG 1952II4934** p1; **SNSB-BSPG 1952II4935** p1; **SNSB-BSPG 1952II4936** p1; **SNSB-BSPG 1952II4937** left m1; **SNSB-BSPG 1952II4938** right m1; **SNSB-BSPG 1952II4939** right mandible with m1; **SNSB-BSPG 1952II4940** left m2; **SNSB-BSPG 1952II4953** left mandible with p2-m3 (this work, Fig. 4.4).

**Measurements.** See S1 Annex.

### Description

**Mandibles.** The mandible is massive with a straight ventral outline in lateral view. The corpus mandibularis becomes regularly larger toward m3. There is no well-preserved angular process. However, the incisura vasorum is not preserved, but does not seem to be pronounced. The angular process and the ramus are very enlarged with a caudal concavity of the ramus edge. The condylar process is high with a very small mandibular incisure. The coronoid process is enlarged and curved. In lateral view, the mandible high abruptly reduces at the beginning of the elongated diastema between p2 and p1. There is a well-developed crest on the dorsal part of the diastema. The caudal edge of the symphysis is at the middle of the diastema. The mandibular foramen is large and located just behind the p1.

**Lower deciduous dentition.** The d2 is double rooted. The d3 is anteroposteriorly elongated and narrow, very similar in shape to p4. The anterior conid is slightly tilted and enlarged. The mesolabial conid is high and central. It is as wide as the tooth with two relatively long posterior parallel crests. The posterolabial conid is large and forms the posterolabial edge of the tooth. There is no mesolingual conid. The transverse cristid is elongated joining the posterolingual corner of the tooth. The posterior valley is anteroposteriorly oriented. The posterior stylid is extremely reduced. The d4 possesses a flat lingual wall, without metastylid or mesostylid. The lingual cuspids are laterally compressed. They possess small and straight cristids. Anterior and posterior cingulids are absent. The ectostylids are present but very weak. The hypoconid and the protoconid are of the same size. However, the posthypocristid is longer than the postprotocristid. The posthypocristid is oblique and joins the postentocristid. The prehypocristid is oblique and leans on the external postprotocristid. The internal postprotocristid is oblique and joins the postmetacristid. The preprotocristid is anteriorly oriented and short. It ends at the base of the anterior fossa. The anterolabial conid and the anterolingual conid are anteriorly oriented and fused at the anterior edge of the tooth.

**Lower permanent dentition.** p1 is conical, tusk-like, and slightly curved. The three other premolars are relatively similar. They are laterally compressed. The anterior conid get larger from p2 to p4 and more and more oblique, enlarging the tooth. The mesolabial conid is high and triangular in occlusal view, as wide as the tooth. Three cristids join on the apex of this cuspid. Labially, the anterolingual cristid is oriented to anterior and reaches the base of the anterior conid. The mesolingual conid is absent. The transverse cristid is very elongated reaching the posterolingual edge of the tooth. The posterlabial cristid, on the labial edge of the tooth, joins the posterolabial conid on the three premolars. This cuspid is increasingly developed from p2 to p4. Thus the posterior valley is anterioposteriorly oriented and deep, posteriorly opened on p2 and p3. The posterior stylid encloses this valley on p4. The p2 shows an unexpected feature in having the back valley closed by a conid (maybe homologous to the posterolingual conid), clearly absent on p3 and p4. On p3, an internal ridge can be observed in the back valley or even a small neocrista also observed in some cases on p4. This neocrista seems to be highly variable. An anterior cingulid is present.

The molars are very selenodont. They become larger from m1 to m3. The trigonid and the talonid are relatively small, the trigonid being larger than the talonid. The preprotocristid is oblique and slightly curved enlarging the trigonid. It joins the well-developed premetacristid. The external postprotocristid is weak on m1 to well marked on m3. The internal postprotocristid, such as the posthypocristid, are transverse. The postprotocristid reaches the base of the postmetacristid and of the preentocristid. The prehypocristid does not join the postmetacristid, between the internal and external postprotocristids. The lingual cuspids are relatively well aligned (the metaconid being a little more lingual), with a small to absent metastylid and entoconulid. The entostylid is absent. The entoconid and the metaconid are slightly labiolingually compressed. The labial cristids are well formed. The high metaconid has a well-marked external postmetacritid, but it does not form a buckle and thus differs from a *Dorcatherium* fold. The entoconid has a deep groove on its posterolabial part, forming a bulge on the labial part of the entoconid. The postentocristid and the posthypocristid are often fused. The postentocristid close the lingual part of the tooth. The quite rounded, in occlusal view, back fossa of m3 is complex and obstructed by a neocristid. This high and complete cristid can cross the back fossa joining the postentocristid, and can separate the back fossa into two fossae. The ectostylids are weak. The anterior and posterior cingulids are well marked.

**Upper deciduous dentition.** The D3 is massive. The labial wall is not flattened. The small anterior cone possesses a labial ridge. The globular paracone is in the centre of the labial wall. The anterolingual crista is connected to the posterior crista of the anterior cone. The protocone only forms a cingulum on the anterior edge of the large metaconule. The posterior fossa possesses a metaconule fold. The metacone possesses a big labial rib with an anterior ridge. The metastyle is well developed. The D4 has clearly a shape that prefigures those of upper molars. However, the protocone is smaller giving a shape more trapezoidal to the tooth. The postprotocrista and the premetaconulecrista do not fuse, the postprotocrista being highly elongated and curved. The parastyle is very enlarged. The paracone and the metacone are not labially in line, the metacone being a little oblique. The mesostyle is located on the metacone anterior extremity. The metastyle is small. No cingulum surrounds the D4.

**Upper permanent dentition.** P3 and P4 has similar lingual wall. The anterior style is globular, salient, and posterior. The anterolabial cone is slightly anterior position and high. The anterolabial cone rib is slightly bulged forming a small rib. The anterolabial crista is straight and aligned. The P3 is sickle shaped in occlusal view. It is slightly laterally compressed. The central lingual cone belongs to the lingual wall forming a small lingual discontinuity on its labial part. On P4, the lingual cone is very enlarged. The fossa is not obstructed by neocristae. The P4 bears well-marked anterolingual and posterolingual cingula, which are almost joining in the transverse axis of the tooth.

The upper molars become larger from M1 to M3. The protocone and the metaconule are similar in size. The preprotocrista joins the base of the straight preprotocrista. The postprotocrista is very elongated and curved ending in parallel of the long and curved premetaconulecrista. The latter is posteriorly forked. The straight postmetaconulecrista joins the base of the postmetacrista forming a smallmetastyle. The metacone and the paracone are not aligned, the metacone being a little oblique. Their cristae are short and straight. The metacone labial rib is weak whereas the paracone one is very prominent with an anterior groove. The mesostyle is large, salient, and prominent, forming a column. The parastyle is well developed at the level of the fusion of the preprotocrista and the preparacrista. There is no ectostyle. The upper molars do not possess cingulum. The fossae are not obstructed by neocristae.

### Discussion

The peculiar shape of the p4, without mesolingual conid, is characteristic of the Tragulidae and Bachitheriidae [42], [54], [61]. The combination of a tusk like p1, a very elongated diastema between p1 and p2 and this structure of the p4 is unique among ruminants and indicate the Bachitheriidae [52], [61]. The differentiation between *Bachitherium* species is mainly based on size differences [20], [64]. Wehrli [69] considered that the size difference corresponds to a sexual dimorphism. But the diastema length [20], the proportions of the postcranial remains [50], [70], and differences in the general shape of the mandible [8], [54], [62], correspond to a species-specific ecomorphology. The p3 and p4 of *Bachitherium* specimens from Gaimersheim1 can possess neocrista within the back valley. Blondel [17] argues that this feature is unique among the Bachitheriidae and is an autapomorphy of the species *B*. *lavocati*. However, similar features have already been observed on *B*. *curtum* and *B*. *insigne*, even if it remains rare [8]. Aiglstorfer *et al*. [71] have already shown the huge variability in the shape of the traguline p4 within one species. The p4 of the Tragulidae *Dorcatherium naui* can lack the cristid forming the lingual edge of the tooth with the entire intermediary possible [71]. The size of the teeth studied here is very similar to those of *Bachitherium curtum* and *B*. *lavocati* [8], [17], [20], [64] (see S1 Annex). However, the diastema length of the specimens from Gaimersheim is longer than those of the small *B*. *curtum*, what clearly corresponds to the diagnosis of *B*. *lavocati* proposed by Sudre [20]. Until now, *Bachitherium lavocati* was only known in the Pech Desse locality [17], [20].

***Bachitherium cf. lavocati** Sudre, 1986*

Fig. 4

### Referred specimen information

**Referred specimen.** *Mailhat* MNHN LIM157 right astragalus (distal trochlea broken) (this work, Fig. 4.1).

### Description & discussion

The specimen MNHN LIM157 presents a similar morphology to those of *Bachitherium* in having trochleas not in line. The edges are slightly different in size and high, the external one being bigger. The plantar crest is absent. There is a large plantar projection of the internal lib of the proximal trochlea. Considering the size provided by Martinez & Sudre [21], this fossil is larger (dorso-plantar height: 12.4 mm and length: 13.3) than specimens of *B*. *viretti* and *B*. *curtum*, and could correspond to very large *B*. *lavocati* or the mean size of *B*. *guiroutensis* (*B*. *insigne* being not described). Looking at the illustration of Martinez & Sudre [21] and Sudre [50], *B*. *curtum*, *B*. *viretti*, and *B*. *guiroutensis* possess trochleas less aligned than on *B*. *lavocati*. Moreover, the lips of the proximal trochlea, in *B*. *guiroutensis* are not symmetrical, contrary to *B*. *lavocati*. Considering all these features, our specimen is most similar to *Bachitherium lavocati*.

*Family **INDET.***

*Genus **“Amphitragulus”** Croizet in Pomel, 1846*

***“Amphitragulus” quercyi** Filhol, 1887*

Fig. 5

### Holotype & referred specimens information

**Holotype.** Bouvrain *et al*. ([72], Fig. 1) has established specimen MNHN Qu4771, a right mandible with the tooth row p2-m3 and ramus partly preserved, as holotype for the species “*Amphitragulus*” *quercyi* (this work, Fig. 5.1), from the Old collection from Quercy (Tertiary, France).

**Referred specimens.** *Old collection from Quercy* **MNHN Qu4771** right mandible with the tooth row p2-m3 and ramus partly preserved (Holotype; [72], Fig. 1; this work, Fig. 5.1). *Balsthal Bännli* **NMS 7685** right mandible with p4-m3 (this work, Fig. 5.2). *Pech Desse* **USTL PDS1312** right mandible with p2-m3 ([17], Fig. 2.4c; this work, Fig. 5.3); **USTL PDS1342** left mandible with p4-m1; **USTL PDS1421** right maxillary with D2-D4 ([17], Fig. 2.4b; this work, Fig. 5.5); **USTL PDS1441** left mandible with p2-m3; **USTL PDS1803** right mandible with p2-m3 ([73], Fig. 14); **USTL PDS2134** right mandible with tooth socket of p1 and tooth row p2-m1 (this work, Fig. 5.4); **USTL PDS2137** left mandible with m1-m3; **USTL PDS2138** right mandible with p3-m1; **USTL PDS2139** left maxillary with P3-M3 ([17], Fig. 2.4a; this work, Fig. 5.6). *Gaimersheim1* **SNSB-BSPG 1952II304** right m2; **SNSB-BSPG 1952II4803** left maxillary with P3-M1; **SNSB-BSPG 1952II4804** right maxillary with D3-M1; **SNSB-BSPG 1952II4811** left mandible with m2-m3; **SNSB-BSPG 1952II4820** left mandible with p4-m1; **SNSB-BSPG 1952II4821** right mandible with m1-m3; **SNSB-BSPG 1952II4844** right mandible with m3; **SNSB-BSPG 1952II4845** right maxillary with M1 & M3; **SNSB-BSPG 1952II4847** left maxillary with D3-M1; **SNSB-BSPG 1952II4851** right mandible with m1-m3; **SNSB-BSPG 1952II4854** left mandible with m1; **SNSB-BSPG 1952II4858** right mandible with m1; **SNSB-BSPG 1952II4865** left mandible with m1-m3; **SNSB-BSPG 1952II4867** right mandible with d4; **SNSB-BSPG 1952II4872** right mandible with m2-m3; **SNSB-BSPG 1952II4873** left m3; **SNSB-BSPG 1952II4878** left mandible with m3; **SNSB-BSPG 1952II4884** right mandible with p3-m2; **SNSB-BSPG 1952II4885** right mandible with p4-m2; **SNSB-BSPG 1952II4891** right M3; **SNSB-BSPG 1952II4892** left M2; **SNSB-BSPG 1952II4894** left mandible with p2-m3; **SNSB-BSPG 1952II4896** right m1-m2; **SNSB-BSPG 1952II4909** right mandible with m2-m3; **SNSB-BSPG 1952II4911** fragmented skull; **SNSB-BSPG 1952II4912** right maxillary with P2-M1 (this work, Fig. 5.7); **SNSB-BSPG 1952II4914** left mandible with p2-m3; **SNSB-BSPG 1952II4917** left p4; **SNSB-BSPG 1952II4920** left maxillary with M1-M3; **SNSB-BSPG 1952II4921** right maxillary with M1-M3; **SNSB-BSPG 1952II4924** left M2; **SNSB-BSPG 1952II4927** right mandible with m1-m3; **SNSB-BSPG 1952II4932** left M3; **SNSB-BSPG 1952II4933** left M3; **SNSB-BSPG 1952II4941** right p4; **SNSB-BSPG 1952II4942** left d3; **SNSB-BSPG 1952II4943** left mandible with d3-m1; **SNSB-BSPG 1952II4944** right maxillary with M2-M3; **SNSB-BSPG 1952II4945** left maxillary with D4-M2; **SNSB-BSPG 1952II4946** left mandible with m1-m3; **SNSB-BSPG 1952II4947** right maxillary with D4-M2; **SNSB-BSPG 1952II4948** right maxillary with P3-M1; **SNSB-BSPG 1952II4949** right maxillary with M1-M3; **SNSB-BSPG 1952II4950** left mandible with p4-m1; **SNSB-BSPG 1952II4951** M2 left; **SNSB-BSPG 1952II4952** right mandible with p3, m2-m3.

**Measurements.** See S2 Annex.

### Description

**Mandibles.** The *corpus mandibulae* is slender. The height below the check-teeth remains more or less the same. The ventral profile is very concave. The long diastema between c and p1 possesses a small constriction in its high. The *incisura vasorum* is weak, blurred, and relatively far from the m3. The *ramus* is slightly backward oriented and large. The condylar process is quite low.

**Lower permanent dentition.** Even if mandibles only possess the one tooth socket of a p1 added to p2 (both teeth being rarely separated, excepted for USTL PDS2134), neither p1 nor p2 is recorded. Blondel [17] described for the locality of Pech du Fraysse that the p1 of “*Amphitragulus*” *quercyi* is small without more details. The degree of molarization rises from p2 to p4. The mesolabial conid is high. It is placed on the anterior half on p2 and becomes larger on p4. The transverse is cristid backward oriented and becomes elongated from p2 to p4, on p4 it ends the mesolingual conid. This conid is high and strong. The presence of anterolingual and posterolingual cristids (on p4) seems variable. They are absent on the specimens of Pech Desse, but present on the specimens of Gaimersheim1 and Balsthal Bännli giving to the anterior valley a different shape. This latter is nearly closed when the anterolingual cristid is present and widely open when absent. The anterior conid on p4 can be absent (Pech Desse) or present (Gaimersheim1 and Balsthal Bännli) forming a forked anterolabial cristid. The posterior conid is becoming larger from p2 to p4, such as the posterior stylid, reaching the posterolingual edge of the p4. On p4, the back valley is ovoid and closed by the partial fusion of the posterolingual conid and the posterior stylid.

On the lower molars, the trigonid and the talonid are closed. The cuspids are bunoselenomorph. The preprotocristid is oblique and joins the premetacristid in the axis of the tooth. The premetacristid is longer on m3 than on m1. The anterior part of m1 is less bulged from m1 to m3, forming a pinched anterior part of the tooth. The internal postprotocristid is becoming longer from m1 to m3, forming a wider trigonid. The external postprotocristid is deep. The oblique prehypocristid is relatively thin and salient. It ends against the postprotocristid. The metastylid is small and salient. The ectostylid is present but becomes smaller from m1 to m3. The talonid is larger from m1 to m3 due to a longer posthypocristid. The posthypocristid is located side by side to the entoconid, but not fused. The lingual cuspids are relatively laterally compressed. They are not in exactly the same axis. As for the entocristids, the premetacristid is longer than the postmetacristid. All the lingual crests are straight. The postentocristid is very short and does not join the posthypocristid. The back fossa is elongated, narrow, and pinched. The hypoconulid forms the edge of this latter. The anterior cingulid is variable, from weak to very strong. The posterior cingulid is quite small.

**Upper deciduous dentition.** The D2 is anteroposteriorly elongated and laterally narrow. On D2 and D3, the small paracone and protocone are situated on the middle of the tooth. They are laterally compressed. On these teeth, the anterior cone has a large rib on its labial part. Instead of a posterior part formed by the mesostyle, the metacone, and a well-developed metaconule are present on D3 and D4. There is an enlarged posterior fossa. Due to the metaconule on D3 and D4, these teeth are enlarged compared to D2. The metaconule is larger on D4 than D3. On D4, the protocone is sickle-shaped in occlusal view with a postprotocrista transversally oriented. The posterior fossa is enlarged with a premetaconulecrista finishing between the paracone and metacone, and an elongated and curved postmetaconulecrista joins a small metastyle. Paracone and metacone are aligned on D4. On D3 and D4, the mesostyle forms a small column. The labial part of the metacone is relatively flat with a weak rib. The paracone on D4 possesses an enlarged labial rib, and anteriorly a globular parastyle, forming a small column. The cingula are weak and there is no entostyle.

**Upper permanent dentition.** The three premolars increase in molarization from P2 to P4. Their labial wall is relatively similar. The anterolabial cone is bulbous on P3, and forms a median rib on P4. The anterior style becomes less pronounced from P2 to P4. There is no posterior style. The lingual cone has a median position. It becomes larger from P2 to P4. On P2, it is small and low. On P4, it is as large as the tooth. The enlargement of this cusp leads to a change of the orientation of the lingual cristae from anteroposterior on P2 to transverse on P4.

The upper molar cusps are relatively bunoselenomorph. They have a short and straight postprotocrista labially oriented. The preprotocrista, relatively straight, joins a well-developed parastyle. The metaconule is slightly reduced and is larger on M3. The premetaconulecrista and the fossae are simple without neocrista. The posterior fossa is tilted, especially on M3. From straight on M1, the postmetaconulecrista becomes sigmoidal on M3, bypassing the metacone. The labial cusps are in the same axis with relatively straight cristae. The paracone labial rib is salient, but without anterior groove. There is no metacone labial rib. The para-, meso-, and metastyles are salient. The lingual cingulum is very weak to absent such as the entostyle.

### Discussion

The recorded structure of the fourth lower premolar is typical for derived stem pecoran and crown pecoran in having a well-developed mesolingual conid and posterior cristid [52], [54]. Moreover, the presence of a tooth socket for the p1 excludes an attribution to *Prodremotherium* or *Mosaicomeryx* [52]. Even if the teeth are not as bunoselenodont as in *Amphitragulus elegans*, the referred specimens have quite primitive features, such as a highly reduced metastylid, a low crown, a small postentocristid, and although laterally compressed, bulged lingual cuspids on lower molars. All of these characteristics exclude an affiliation to either *Dremotherium* or *Bedenomeryx* [8], [74]. Furthermore, the extremely small size of “*Amphitragulus” quercyi* is a distinguishing feature for the latest Oligocene ruminants of Europe [16], [17], [72], [75].

*Genus **Dremotherium** Geoffroy Saint-Hilaire, 1833*

***Dremotherium cf. guthi** Jehenne, 1987*

Fig. 6

**Fig 6:**
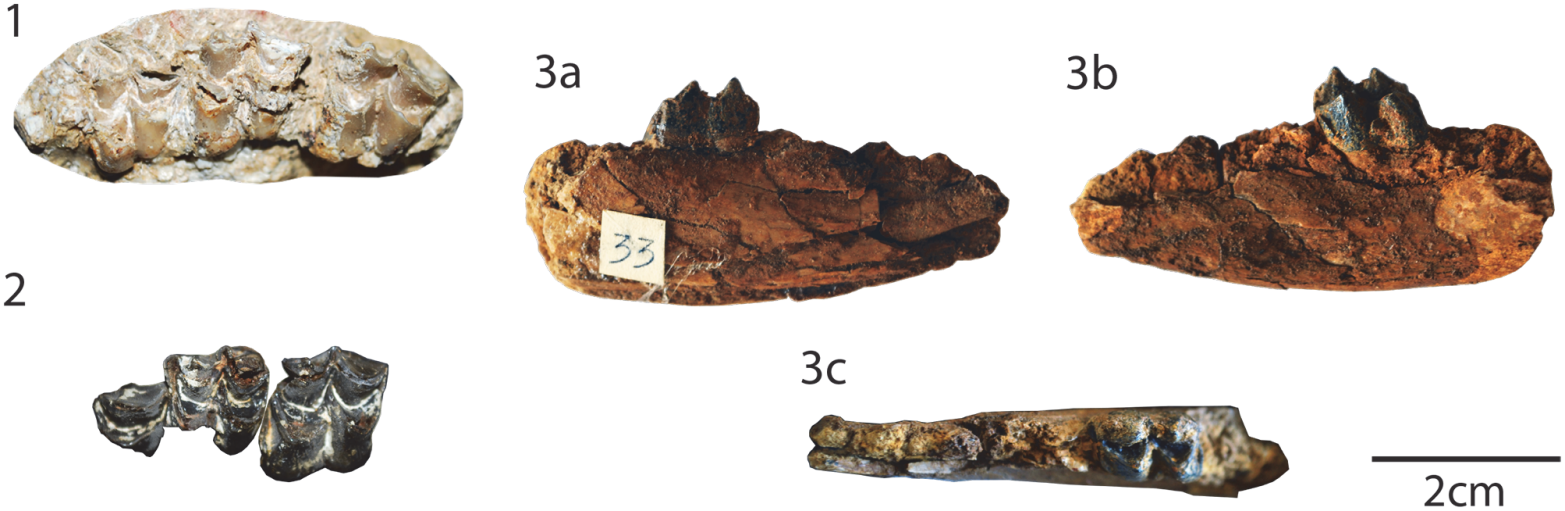
Specimens of *Dremotherium* cf. *guthi* and *Babameryx engesseri*. *Dremotherium* cf. *guthi* from *Gaimersheim1*: **1** left maxillary bone with the tooth row M1-M3 (SNSB-BSPG 1952II4855). from *Mailhat*: **2** left P4-M2 (MNHN LIM154). *Babameryx engesseri* from *Gaimersheim1*: **3** left fragmentary mandible with m2 (SNSB-BSPG 1952II4818), (a) lingual, (b) labial, and (c) occlusal views. The scale bar is 2 cm.

### Holotype & referred specimens information

**Holotype.** Jehenne (1987) has appointed the specimen IPHEP LM.1969.MA.40 as holotype. This is a partial right maxillary bone with P3-M3 ([76], Fig. 2), from La Milloque (MP29, France).

**Referred specimens.** *Mailhat* **MNHN LIM154** left P4-M2 (this work, Fig. 6.2); *Gaimersheim1* **SNSB-BSPG 1952II4855** left maxillary with M1-M3 (this work, Fig. 6.1); **SNSB-BSPG 1952II4870** right m2.

**Measurements.** MNHN LIM154 P4 (9.2x?), M1 (11.0x?), M2 (12?x14.1); SNSB-BSPG 1952II4855 M1 (x), M2 (x), M3 (x).

### Description

**Lower permanent dentition.** The lower molar is very badly preserved. However, this big ruminant possesses a fully selenomorph trigonid. The cristid are elongated and the cuspids are high with the enamel slightly pleated. The metastylid is well developed.

**Upper permanent dentition.** The P4 is badly preserved. All the lingual part is missing. The anterolabial cone possesses a weak labial rib. The anterior stylid is broken. The posterior one is small but present. No neocrista obstructs the fossa. The quadratic upper molars in occlusal view bear a well-developed metaconule slightly smaller than the protocone. The cusps are very selenomorph. The preprotocrista and the postmetaconulecrista are labially oriented and quite straight. The postprotocrista is long, highly curved, and labially oriented. It can be completely isolated (SNSB-BSPG 1952II4855) or fused (MNHN LIM154) to the premetaconulecrista. The latter ends at the axis of the mesostyle. The posterior basin can possess a small metaconule fold on M2, larger on M3. The paracone, possessing a paracone rib, and the metacone, labially flat and concave, are aligned. The mesostyle and parastyle are well developed. They form rounded small columns. The mesostyle is larger from M1 to M3. The entostyle is weak or absent. There is neither a lingual cingulum surrounding the protocone nor a metastyle forming a well-developed cuspid. However, small anterior and posterior cingula are present.

### Discussion

This ruminant is relatively advanced in being well-selenodont and having no lingual cingula in upper molars, always present in *Lophiomeryx*, *Gelocus*, *Iberomeryx*, *Mosaicomeryx*, and *Babameryx* [42], [52], [54], [61–63], [73]. Contrary to *Bachitherium*, this species lacks an anterior groove on the very large labial paracone rib [8], [42], [61]. The studied fossils are more similar to the advanced stem Pecora and to the primitive crown Pecora (such as *Amphitragulus*, *Dremotherium*, *Bedenomeryx*). The enamel is slightly pleated, whereas it is generally smooth in *Prodremotherium*. Moreover, the size of these teeth is significantly larger than in *Prodremotherium* from the old Quercy collections [64] and Pech Desse [17]. The teeth are more selenomorph than in “*Amphitragulus*” and *Bedenomeryx* [8], [68], [77] and the parastyle is not that anteriorly projected than observed on “*Amphitragulus*” *feningrei* [62], [78], [79]. Looking at the neodiagnosis proposed by Mennecart [8], these fossils include all indicating features of *Dremotherium*. The currently defined species in Europe are *D*. *feignouxi* (type species) and *D*. *guthi* [8], [76], [80]. The latter is slightly smaller than the type species [76], similar to the fossils described here. *D*. *feignouxi* only occurred during the Agenian (MN1-MN2; [80]), whereas *D*. *guthi* lived during the latest Oligocene (MP28-MP30; [76]). However, in absence of additional well-preserved material, a definitive assignation to the species is not possible.

*Genus **Babameryx** Mennecart, 2012*

***Babameryx engesseri** Mennecart, 2012*

Fig. 6

### Holotype & referred specimen information

**Holotype.** NMO-K11/15 is a left M1 ([16], Fig. 6.2), from Rickenbach (MP29, Switzerland).

**Referred specimen.** *Gaimersheim1* **SNSB-BSPG 1952II4818** left fragmentary mandible with m2 (this work, Fig. 6.3).

**Measurements.** SNSB-BSPG 1952II4818 m2 (10.0x6.9).

### Description & discussion

The *Babameryx engesseri* specimen clearly differs from all other Gaimersheim1 ruminants’ specimens. This specimen is much larger than “*Amphitragulus*” *quercyi* [8], [17], [75] (S2 Annex), and to a lesser extent bigger than *Bachitherium lavocati* [17], [20] (S1 Annex), but smaller than *Dremotherium guthi* [8], [76]. Its size and morphology are in accordance with other described *Babameryx* specimens. It possesses a regularly concave ventral outline of the mandible. In *Bachitherium*, the lower outline of the mandible is always straight [20], [42], [54], [62]. Moreover, the teeth of *Babameryx* are a little more bunodont than those of *Bachitherium* and *Dremotherium* [16] in having more bulged lingual cuspids. The trigonid and talonid are reduced due to transverse internal postprotocristid and posthypocristid. Contrary to *Bachitherium*, the postentocristid and the posthypocristid do not fuse together [16], [42]. The posthypocristid reaches the lingual part of the tooth. The lingual cristids are small in comparison to those of *Dremotherium* giving to the molars of *Babameryx engesseri* a sharper and more bunodont aspect [16], [76]. An external postprotocristid is present. The metastylid is very reduced. The anterior and posterior cingula are big and the ectostylid is well developed. This is the oldest occurrence of the species [16].

## Discussion

### About the age of the Mailhat locality

**Fig 7:**
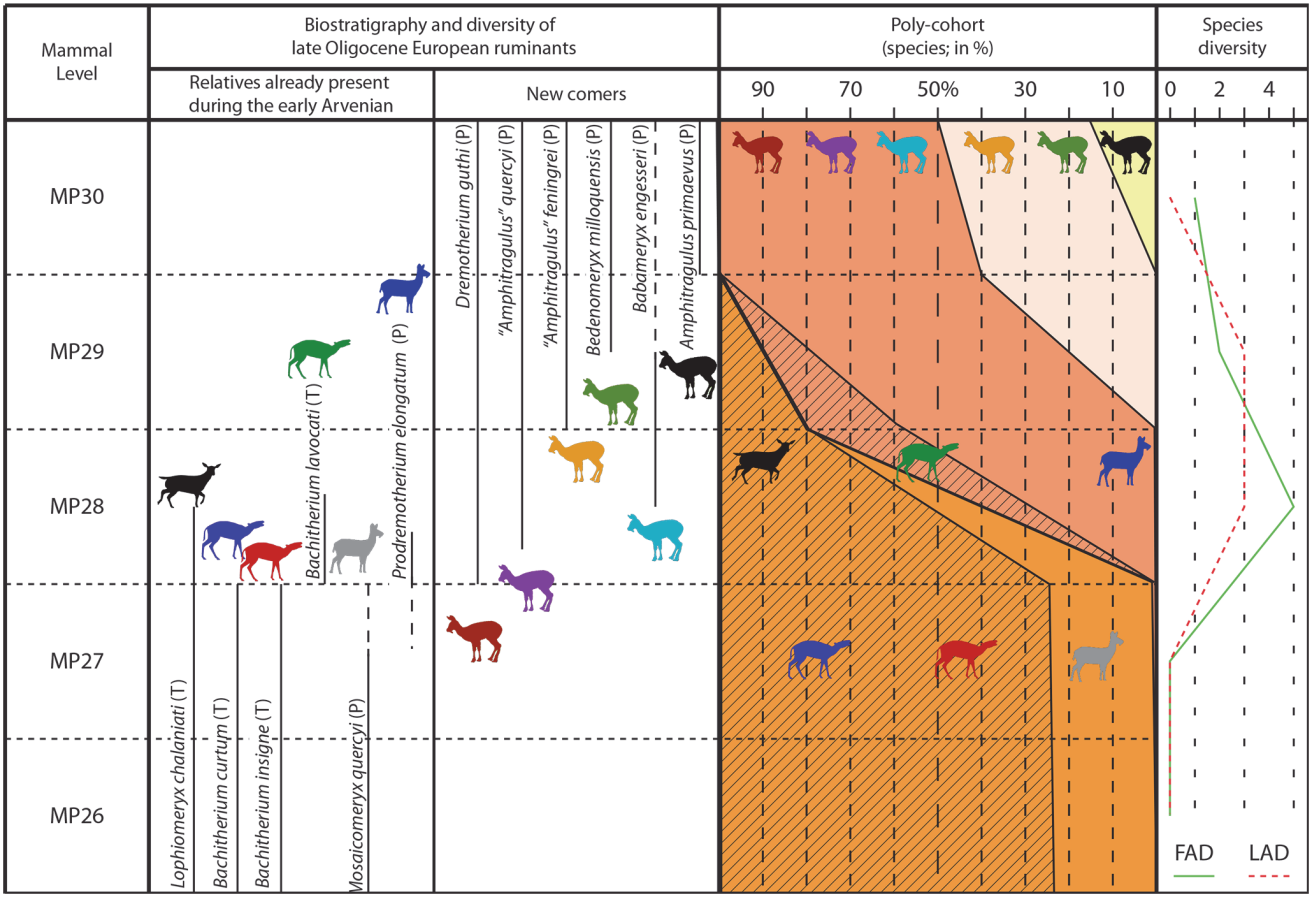
Biostratigraphy and evolution of Western Europe latest Oligocene ruminant (origination/extinction curves and survivorship poly-cohort [%]). (ruminant data derived from [16], [8], [17], [13], [23], [28], [29], [47], [84] have been modified according to the results from this study; the biostratigraphy is based on [9]). (T) Tragulina; (P) Pecora. In the Polycohort area, the stripes represent the Tragulina.

**Fig 8:**
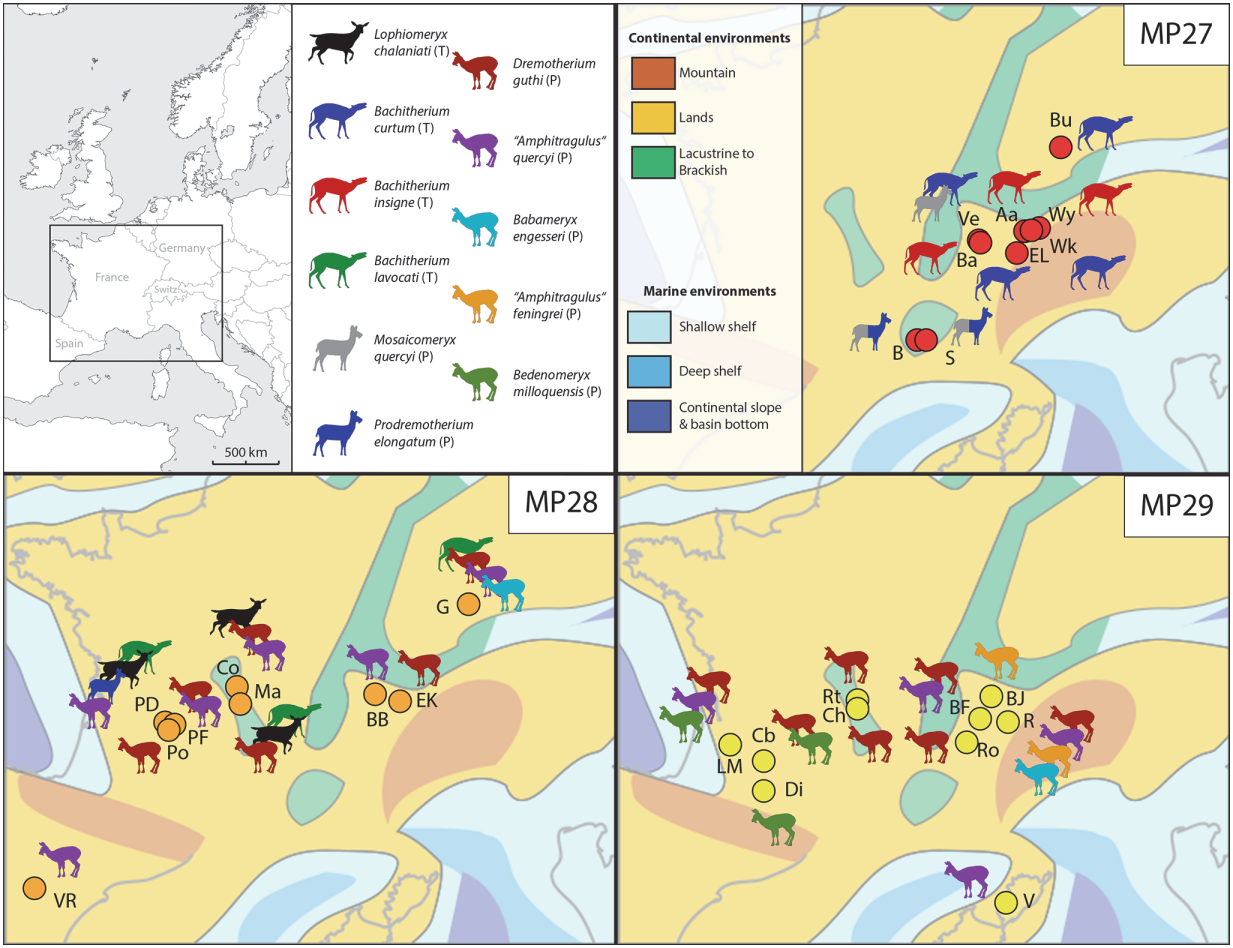
Late Oligocene ruminant paleobiogeography of Western Europe. Aa Aarwangen; B Boujac; Ba La Barboille; BB Balstahl Bännli; BF Brochene Fluh 19/20; BJ Basel St Jakob; Bu Burgmagerbein1; Cb Comberatière; Ch Les Chauffours; Co Cournon; Di Dieupentale; EK Ebnat Kapel; EL Eriz Lösenegg; G Gaimersheim1; LM La Milloque; Ma Mailhat; PD Pech Desse; PF Pech du Fraysse; Po Portal; R Rickenbach; Ro La Rochette; Rt Romagnat; S Sarèle; Ve Veyron2; V Vazzio; VR Vivel del Rio; Wk Wolfwil kennisbännli; Wy Wynau iii and Wynau V. (T) Tragulina; (P) Pecora. Considered data based on references given for Fig. 7. Paleogeographical map of the latest Oligocene had been modified from [85] and [86].

Due to the absence of micromammals, the Mailhat locality is poorly dated. Hugueney [23] proposed an age within MP23 and MP28, based on the occurrence of *Anthracotherium magnum*, *Plesiomeryx* sp. (*Cainotherium* sp. of [23], pers. obs.), and *Lophiomeryx chalaniati*. However, the large mammals fauna from this locality gives an age comprised between MP25 and MP28 (*Anthracotherium magnum* MP25–MP29 [9], [62], [81], [82]; *Plesiomeryx* sp. MP21–MP28 [9], [83]; *Lophiomeryx chalaniati* MP24–MP28 [8], [29], [51]). Moreover, the description of new ruminants in this locality greatly improves the precision in the age of this locality. *Dremotherium guthi* has only been described between MP28 and MP30 to date [62], [76] (Figs. 7 & 8). The only co-occurrence of this species with a *Lophiomeryx* in Europe is in Cournon (France), a locality dated to MP28. It is interesting to note that these two localities are very closely situated in the Limagne graben (Fig. 1). Moreover, the only known localities containing *Bachitherium lavocati* (Pech Desse and Gaimersheim1) are both dated to MP28 based on micromammals [17], [20] (Figs. 7 & 8).

In conclusion, it is suitable to admit, if all specimens belong to a same fossiliferous layer, that the Mailhat fauna represents the biozone MP28. Considering the presence of non-Pecoran ruminants, absent in younger localities (such as Pech du Fraysse and Ebnat Kappel), Mailhat is older than Pech du Fraysse, but younger than Pech Desse.

### Biostratigraphy

*Lophiomeryx* and *Bachitherium* are typical ruminants from the European Oligocene fauna. These genera appeared closely after the “Grande Coupure” event, during MP22 [8], [9], [87] and are probably part of the Asiatic immigrants (*Lophiomeryx* has been found in Eocene Asiatic deposits, e. g. [63], [66], [67], [88], [89]). The last occurrence of these two genera in Europe corresponds to MP28. *Lophiomeryx chalaniati* is recorded in Cournon [23], Pech Desse [17], [29], and Mailhat [23], [28], [29]. Its occurrence interval is comparably long (from MP24 to MP28; [9], [17], [20]; Fig. 7). The remains of these species are extremely rare in latest Oligocene localities: only two specimens in Cournon [29] and one mandible fragment in Pech Desse [17], [29], (see Fig. 3.1) were identified without any doubt. Furthermore, Blondel [17] documented 33 astragali from Pech Desse, according to the data of Martinez & Sudre [21]. However, no postcranial remains from Pech Desse were properly described and identified. Up to now, *Bachitherium lavocati* was only known in Pech Desse [17], [20]. The Gaimersheim1 specimens confirm the latest Oligocene occurrence of this species. It seems to be highly informative for biostratigraphy being only present in old MP28 localities (Pech Desse, Gaimersheim1; [22], and probably Mailhat; Figs. 7 & 8). It has never been observed in association with the classical Oligocene Bachitheriidae: *B*. *curtum* and *B*. *insigne*, of which the last occurrences are documented during MP27 [8], [9], [13] (Figs. 7 & 8). The occurrence of *Prodremotherium elongatum* is also highly temporally restricted. It only occurred undoubtedly during the base of MP28 in Pech Desse, and maybe in the late MP27 [52], [17], [84] (Figs. 7 & 8). This taxon seems to take the place of its close relative *Mosaicomeryx quercyi*, which lived in Europe between MP25 and MP27 [13], [52] (Figs. 7 & 8). An Asiatic origin for *P*. *elongatum* can be assumed, since this taxon has been already mentioned from Early Oligocene Asiatic localities (Kazakhstan, [66]; Mongolia, [90]; Georgia, [91]).

There is an acceptance that *Lophiomeryx* is a member of Tragulina and *Prodremotherium* is a basal stem pecoran, whereas the phylogenetic position of *Bachitherium* within the Tragulina is still debated [52], [61]. The other latest Oligocene newcomers (“*Amphitragulus*” *quercyi*, *Dremotherium guthi*, *Babameryx engesseri*, and a little latter “*Amphitragulus*” *feningrei* and *Bedenomeryx milloquensis*; see Figs. 7 & 8) are considered to be more derived stem Pecora, or even crown Pecora [52], [62], [92]. They all possess a highly derived lower fourth premolar structure being massive and possessing a well-developed mesolingual conid. In *Bachitherium*, *Lophiomeryx*, and *Prodremotherium* this is not the case [52]. “*Amphitragulus*” *quercyi* is the first representative of this clade in Europe. It appeared at the same time as *Bachitherium lavocati* and *Prodremotherium elongatum* in Pech Desse. “*A*.*” quercyi* has been listed in Boningen (type locality for MP27; [93]) this could be the oldest record for this species. However, the referred specimens of this locality are too badly preserved to be identified as Pecora ([47], personal observation). “*A*.” *quercyi* and *Dremotherium guthi* were already described from Asia [67], [90]. The immigration of this cohort continued until the beginning of the Miocene where a large diversity of ruminants occurred with no less than seven different genera (*Amphitragulus*, *Andegameryx*, *Bedenomeryx*, *Dremotherium*, *Friburgomeryx*, *Oriomeryx*, *Pomelomeryx*; [8], [9], [62], [80]. The appearance of derived Pecora in Europe coincides with the temporary disappearance of the Tragulina (Fig. 7).

### Causes and consequences of the Ruminant fauna renewal

**Environmental changes.** The latest Oligocene corresponds to a major faunal renewal among the mammals, such as the rodents [15], the terrestrial cetartiodactyls [9], [17], [18], and the perissodactyls [9], [19], [49], but also among the reptiles, such as the booids [14], and the plants [12], [13]. This crisis is named “*Microbunodon* Event” [9]. Indeed, the time interval MP27-MP30 (ca. 24.3 and 22.9, [94]) is marked by huge global changes. It began with the Late Oligocene Warming and ended with the Mi1 glaciation [94], which characterizes the Oligocene-Miocene transition. The seawater temperature increased by 2 to 4°C and the temperature on continents by nearly 10°C [6], [10], [15]. This ecological turnover can also be correlated with environmental and sedimentological changes, well marked in the Swiss Molasse Basin. The USM1 deposits, characterised by flood plain deposits (“Molasse Rouge” and the “Untere Bunte Mergel”), change into the USM2 deposits (coal in La Rochette, and gypsum deposits in the Jura Molasse) [82], [86], [94], [95]. This peculiar configuration of wet and dry environment in close area is probably due to paleotopography [82]. The original altitude of the La Rochette mines is estimated at 500 m [82]. The Late Oligocene Warming, associated with the Alpine orogeny, gave birth to changes in the sedimentary processes (flood plain deposits to evaporitic processes) and probably changes in the climate (more arid, warmer, with seasonality) and vegetation cover [4], [12], [82].

**Regionalism intensification.** The biostratigraphy proposed by the rodents indicates that Pech du Fraysse and Gaimersheim1 are contemporaneous [37]. However, the ruminants are different by the abundant presence of *Dremotherium guthi* in the French locality, and of *Bachitherium lavocati* in the German one. This difference could be linked to different paleoenvironments, *D*. *guthi* living in more open environment [16]. Considering the cenogram analyses, Southern France and Switzerland communities clearly define a wooded savannah environment [16], [96]. The area of Gaimersheim1 remained an environment relatively similar from the rest of the Arvenian [96]. Gaimersheim1 could have been a refuge area with more wooded environment in an active tectonic context (Fig. 8). Similar observations have been noticed during the latest Oligocene and earliest Miocene, using cenogram analysis [97] and the postcranial skeleton of ruminants [9], [98]. Moreover, different environmental conditions could explain the regionalism of *Bedenomeryx*, only located in the arid South-western France, and “*Amphitragulus*” *feningrei* only found in more central Europe (Fig. 8). “*Amphitragulus*” *quercyi* and *Dremotherium guthi* invaded the entire Europe from a precocious immigration since MP28.

**Synecological changes.** Considering our analysis, we observe, such as Blondel [17] and to a lesser extent Jehenne & Brunet [99] that the surviving taxa of the Early Oligocene first disappeared in Europe during the Late Oligocene Warming; they are replaced by species from the same family or closely related (*Bachitherium* and *Prodremotherium*; see Figs. 7 & 8). Then these taxa totally disappeared and more derived ruminants rose (see Figs. 7 & 8). The paleoecology of the other mammals also changed. Considering the postcranial remains, the mid-Oligocene species are characteristic of wooded environments (*Bachitherium curtum*, *B*. *inigne*, and *Mosaicomeryx quercyi*), contrary to the latest Oligocene ones (such as *Prodremotherium elongatum*, *Bachitherium lavocati*, *Dremotherium guthi*), which lived in more open habitats [9], [52], [62], [70]. Paleoecological changes have also been observed among other mammal groups. Looking at the rodents, the ratio of omnivorous, soft plant eaters, and very hypsodont burrowing the ground increase, whereas those probably adapted to arid environment disappeared in Quercy [15]. Considering the rhinoceros, the cursorial and mediportal locomotion types are dominating after the “*Microbunodon* Event”, while cursorial type with rare graviportal occurred before [9].

The end of the Oligocene is marked by the appearance in Europe of a seasonality characterized by a dry season [13], [16]. Accordingly, these new environmental conditions led to a major faunal renewal between the primitive families and the more derived Pecora, which possessed a more efficient metabolism and were better adapted to dry habitats. By comparing the fossil Tragulina (Bachitheriidae and Lophiomerycidae) with the living Tragulidae, Janis [100] considered that the primitive European Tragulina are mainly distinguished by forestomach fermentation with a less efficient ruminating system during the Oligocene. Nowadays, the Tragulidae only live in the tropical forests of Africa and South-East Asia [101], [102]. The opening of the environment with fewer forests during the latest Oligocene and the appearance of the seasonality could have been fatal for these primitive ruminants and induced the total renewal of the ruminant fauna during a very short time interval (Figs. 7 & 8).

## Conclusion

The latest Oligocene (between MP27 and MP30) is a period of tremendous diversity increase of ruminants in Europe with at least 12 different species from 10 different genera. During the middle Oligocene, only 3 different genera occurred with a maximum of 5 synchronous species. All the Late Oligocene species are only partly contemporaneous and thus their successive occurrence allows for a precise biostratigraphy of the latest Oligocene. For example, MP27 is the last appearance datum of *Bachitherium curtum* and *B*. *insigne*. MP28 is the last appearance datum of *Lophiomeryx*, the only occurrences of *B*. *lavocati* and *Prodremotherium elongatum*, and the first appearance datum of the more derived “*Amphitragulus*”, *Dremotherium*, and *Babameryx*. This diversity increases until the Early Miocene. *Bachitherium lavocati* was only known in the Pech Desse locality, in France. The occurrence of this species in Gaimersheim1, Germany and Mailhat, France (based on the association of *Lophiomeryx* and *Dremotherium*) during MP28 confirms its usefulness for the characterisation of this biozone.

The time interval of MP28 is very important for the evolution of mammals of Western Europe, and especially of the ruminants. Local species have been replaced by Asiatic migrants. This renewal seems to occur in two phases: first resident, non-pecoran and basal pecoran taxa from the Oligocene gave way to new species phylogenetically related to the previous ones (*Bachitherium lavocati* and *Prodremotherium elongatum*). However, these newcomers only occurred during a very short time interval and then were rapidly replaced by more derived pecorans. Climatic variations between the Western coast, influenced by the Atlantic ocean, and the more central Europe could provided faunal regionalism during the latest Oligocene and earliest Miocene. Moreover, the general creation of open savannahs and the appearance of the seasonality could have been fatal for primitive ruminants. It induced the total renewal of the ruminant fauna at the family level during a very short time interval marked by the total disappearance of the Tragulina in Europe until the late Early Miocene. This period corresponds to a major faunal event considering the European ruminant history: the “*Microbunodon* Event”. In our present day context of climate changing, where migrations of species are already observed, it is highly interesting to consider the past to prevent future extinctions.

## Supporting Information

S1 Annex *Bachitherium lavocati* tooth size in mm. (PDF)

S2 Annex *“Amphitragulus” quercyi* tooth size in mm. (PDF)
