# Supplementary material for: The European Ruminants during the “Microbunodon Event” (MP28, Latest Oligocene): Impact of Climate Changes and Faunal Event on the Ruminant Evolution
Source: PLoS One. 2015 Feb 18;10(2):e0116830. doi: 10.1371/journal.pone.0116830 (PMC4334963; doi:10.1371/journal.pone.0116830)
Supplement: S1 Annex — (PDF) [file pone.0116830.s001.pdf]

Annexe1 *Bachitherium lavocati* tooth size

	p1		p2		p3		p4		m1		m2		m3		p2-4	m1-3
BSPG 1952II4800			7,1		7,9											
BSPG 1952II4801									8,3	5,5						
BSPG 1952II4802							7,9	4	7,2	5,1	8,2	5,5		6		
BSPG 1952II4810					8,5	3,6										
BSPG 1952II4812											8,6	5,8	13	5,7		
BSPG 1952II4813					7,9	3,3	8,7	4,2								
BSPG 1952II4814			5,5	2,6												
BSPG 1952II4816													14,2	6,5		33
BSPG 1952II4817									8,6	5,5	9,7	6,5	15,2	6,3		35,2
BSPG 1952II4819											8,9	6,2	12,7	6,2		
BSPG 1952II4822									7,2	6,1	8,5					
BSPG 1952II4823					9,1	3,5	9,7	5,3								
BSPG 1952II4824					8,1	3,8	9,3	4,1								
BSPG 1952II4825											8,6		11,5			
BSPG 1952II4826					7,3	3	7,1	3,7	7,3	5,1						
BSPG 1952II4833									8	5,2	8,6	6,9				
BSPG 1952II4834									8,4	5,6	9,5	6,2				
BSPG 1952II4835									7,4	5,1						
BSPG 1952II4837													14,9	7		
BSPG 1952II4839							8,2	4,2	7,8							
BSPG 1952II4840									7,4	5,2						
BSPG 1952II4843											9,1	6,4				
BSPG 1952II4846									7,7	5,6	8,9	6,5				
BSPG 1952II4848														7,8		
BSPG 1952II4849									8,5	6,4	9,5	7				
BSPG 1952II4850													15,6			
BSPG 1952II4852			7,5		8,6		9					6,8	15,4	6,8		36,8
BSPG 1952II4853													14,7	6,7		
BSPG 1952II4861											9,8	6,9				
BSPG 1952II4866													14,3	6,5		
BSPG 1952II4869							7,1	3,7	7,5							
BSPG 1952II4871											9,4	6,8				
BSPG 1952II4875											9,7	6,7				
BSPG 1952II4876													15	7,2		
BSPG 1952II4877													13,8	6,4		
BSPG 1952II4879													14	6,2		
BSPG 1952II4881			6,4		7,8		8,4	4,3	7,9	5,4	9,1	6,2			22,8	
BSPG 1952II4886										5,8						
BSPG 1952II4887					7,5	3,8										
BSPG 1952II4893															22,8	
BSPG 1952II4895									7,3	5,3	8,6	6,1	13,9	6,7		31,2
BSPG 1952II4905											9,2	6,5	13,9	6,5		30,6
BSPG 1952II4906					5,9		7,6									
BSPG 1952II4907						3,3					8,6					
BSPG 1952II4908			7,1	2,8	8,4	3,6	9,3	4,5	8,5	6,3					25	29,6
BSPG 1952II4910									8,9	6,5	10	7,3	14,5	7,2		36,7
BSPG 1952II4936	4	3,5														
BSPG 1952II4937									8,6	5,8						
BSPG 1952II4938									8,7	6						
BSPG 1952II4939									7,8	5,3						
BSPG 1952II4940											8,8	6,2				

Annexe1 *Bachitherium lavocati* tooth size

	P2		P3		P4		M1		M2		M3		M1-3
BSPG 1952II4827			8,6	6,5	5,2	9							
BSPG 1952II4831							8,6	10,4					
BSPG 1952II4832								10,2					
BSPG 1952II4836										9,5			
BSPG 1952II4856											9,5	10,6	
BSPG 1952II4859							8,5	9,5					
BSPG 1952II4860									9,3	11,1			
BSPG 1952II4869	7,9	3,6											
BSPG 1952II4874											10,5	12	
BSPG 1952II4882			8,5										
BSPG 1952II4902			8,9	7,5	7	9,8			10,1	11,2	10,6	11,9	28,5
BSPG 1952II4902			9,6	8	7	9,6	8,8	10,5					
BSPG 1952II4904											10,2	12	26,7
BSPG 1952II4916							9,1	10,2					
BSPG 1952II4918					6,6	8	7,4	9,6					
BSPG 1952II4919											10	10,8	
BSPG 1952II4922									9		9,9	11,9	25,9
BSPG 1952II4923					7,1	8,5			9,4	10,8	9,3	11,3	25,7
BSPG 1952II4925							6,8	9,2	9	10,2	10,5	12	26
BSPG 1952II4926							7,7	10,2	9,2	11,8	10,3	12	26,8
BSPG 1952II4928									8,5	10,4			
BSPG 1952II4929											10,2	12,6	
BSPG 1952II4930							7,8	10,4					
BSPG 1952II4931											10,1	10,3	

	d3		d4	
BSPG 1952II4828			10	5,2
BSPG 1952II4829				4,7
BSPG 1952II4830			11,3	4,8
BSPG 1952II4833	7,5	3,4	10,1	4,7
BSPG 1952II4834				4,9
BSPG 1952II4841	7,2		9,5	4,2
BSPG 1952II4862	8,1			
BSPG 1952II4863	7,4	3		
BSPG 1952II4864	8,2	3,2		
BSPG 1952II4889				4,6
BSPG 1952II4890	8	3		
BSPG 1952II4895				4,5

	D3		D4	
BSPG 1952II4805	9,5	7,1	8,4	8,9
BSPG 1952II4806	9,6	6,7	7,9	8,6
BSPG 1952II4807	9,2	6,6		
BSPG 1952II4808	8,9	6		
BSPG 1952II4809	9,7	6,7		
BSPG 1952II4857	9,5	7	8,2	8,7
BSPG 1952II4916	9,9	7,2		

Annexe1 *Bachitherium lavocati* tooth size

	p1		p2		p3		p4		m1		m2		m3		p2-4	m1-3
nomber of specimens	1	1	5	2	11	8	11	9	20	19	20	18	16	16	3	7
mean	4	3,5	6,72	2,7	7,9	3,5	8,4	4,2	7,9	5,6	9,1	6,5	14,2	6,6	23,5	33,3
maximum			7,5	2,8	9,1	3,8	9,7	5,3	8,9	6,5	10	7,3	15,6	7,8	25	36,8
minimum			5,5	2,6	5,9	3	7,1	3,7	7,2	5,1	8,2	5,5	11,5	5,7	22,8	29,6
Standard Deviation			0,8	0,1	0,8	0,3	0,9	0,5	0,6	0,5	0,5	0,4	1,1	0,5	1,3	2,9

	P2		P3		P4		M1		M2		M3		M1-3
nomber of specimens	1	1	4	3	5	5	8	8	7	7	11	11	6
mean	7,9	3,6	8,9	7,3	6,58	8,9	8,1	10,0	9,2	10,7	10,1	11,6	26,6
maximum			9,6	8	7,1	9,8	9,1	10,5	10,1	11,8	10,6	12,6	28,5
minimum			8,5	6,5	5,2	8	6,8	9,2	8,5	9,5	9,3	10,3	25,7
Standard Deviation			0,5	0,8	0,8	0,7	0,8	0,5	0,5	0,7	0,4	0,7	1,0

	d3		d4		D3		D4	
nomber of specimens	6	4	4	8	7	7	3	3
mean	7,7	3,2	10,2	4,7	8,2	5,6	6,9	6,1
maximum	8,2	3,4	11,3	5,2	9,9	7,2	8,4	8,9
minimum	7,2	3	9,5	4,2	8,9	6	7,9	8,6
Standard Deviation	0,4	0,2	0,8	0,3	0,3	0,4	0,2	0,1
