# Supplementary material for: The European Ruminants during the “Microbunodon Event” (MP28, Latest Oligocene): Impact of Climate Changes and Faunal Event on the Ruminant Evolution
Source: PLoS One. 2015 Feb 18;10(2):e0116830. doi: 10.1371/journal.pone.0116830 (PMC4334963; doi:10.1371/journal.pone.0116830)
Supplement: S2 Annex — (PDF) [file pone.0116830.s002.pdf]

Annexe2 *“Amphitragulus” quercyi* tooth size

	p2		p3		p4		m1		m2		m3		p2-4	m1-3
BSPG 1952II4811									7,9	5,9				
BSPG 1952II4820					7,4		7,9							
BSPG 1952II4821							7,9		8,2		12			
BSPG 1952II4844											12,3			
BSPG 1952II4851							8		7,6		11,6		26	26
BSPG 1952II4854								5,2						
BSPG 1952II4858							8	5,2						
BSPG 1952II4865									7,8	6,9	11,5	5,8	26,9	26,9
BSPG 1952II4872										5,8				
BSPG 1952II4873											11,7	6		
BSPG 1952II4878											10,2	5,3		
BSPG 1952II4884					7,3	4								
BSPG 1952II4885					7,2	3,9			8,2	6				
BSPG 1952II4894	5,3		6,6		7		8,3							
BSPG 1952II4909									7,6		10,8			
BSPG 1952II4914	6,2		7		7,2		7,5		8,5		11,3		27,5	27,5
BSPG 1952II4917						3,6								
BSPG 1952II4927									7,3	5,8	12,2	5,9	27,2	27,2
BSPG 1952II4941					6,7	3,7								
BSPG 1952II4943							9							
BSPG 1952II4946							7,8	4,9	8,2	5,5		6,4	29	29
BSPG 1952II4950					7,5		8,5	5,7						
BSPG 1952II4952			6,5	3,1	7,3	4			8,5	5	12,8	5,4		

	P2		P3		P4		M1		M2		M3		M1-3
BSPG 1952II4803			7,2	6,2	4,8	7,1	7,4	8,8					
BSPG 1952II4804							8,8						
BSPG 1952II4845							7,4				8,3		
BSPG 1952II4847							8,5						
BSPG 1952II4891											7,8	7,9	
BSPG 1952II4892									7,9	8,7			
BSPG 1952II4911					6,5	7,2	7,8	9,2	8,4	9,5			
BSPG 1952II4912	6,4	4,5	6,7	6	5,3	7,4	7,5	8,3					
BSPG 1952II4920									8,5	10,1	8	9,4	
BSPG 1952II4921							6,7	9	8,4	9,5	8	8,8	23,5
BSPG 1952II4924									7,9	9,5			
BSPG 1952II4932											9,1	10,9	
BSPG 1952II4933											7,8	9,9	
BSPG 1952II4944									9,3	9,5	8,3	9,5	
BSPG 1952II4945							8,3	9,7	9,2	9,5			
BSPG 1952II4947							6,5	8,2	8,1	8,5			23,5
BSPG 1952II4948			7,6										
BSPG 1952II4949									7,8		8,5		
BSPG 1952II4951									8	9,4			

	d3		d4	
BSPG 1952II4867				4,7
BSPG 1952II4942	6,9	2,5		
BSPG 1952II4943	7,3		10,3	

	D3		D4	
BSPG 1952II4804	8,1	6,3	7,1	7,4
BSPG 1952II4847	7,5	7,2	6,5	7,5
BSPG 1952II4945			5,9	7,6

Annexe2 *“Amphitragulus” quercyi* tooth size

	p2		p3		p4		m1		m2		m3		m1-3
nomber of specimens	2	0	3	1	8	5	9	4	10	7	10	6	5
mean	5,7		6,7	3,1	7,2	3,8	8,1	5,25	8,0	5,8	11,6	5,8	27,3
maximum	6,2		7	3,1	7,5	4	9	5,7	8,5	6,9	12,8	6,4	29
minimum	5,3		6,5	3,1	6,7	3,6	7,5	4,9	7,3	5	10,2	5,3	26
Standard Deviation	0,6		0,3		0,2	0,2	0,4	0,3	0,4	0,6	0,7	0,4	1,1

	P2		P3		P4		M1		M2		M3		M1-3
nomber of specimens	1	1	3	2	3	3	9	6	10	9	8	6	2
mean	6,4	4,5	7,2	6,1	5,5	7,2	7,6	8,9	8,3	9,3	8,2	9,4	23,5
maximum			7,6	6,2	6,5	7,4	8,8	9,7	9,3	10,1	9,1	10,9	23,5
minimum			6,7	6	4,8	7,1	6,5	8,2	7,8	8,5	7,8	7,9	23,5
Standard Deviation			0,4	0,1	0,9	0,1	0,8	0,6	0,5	0,8	0,4	1,0	0

	d3		d4		D3		D4	
nomber of specimens	2	1	1	1	2	2	3	3
mean	7,1	2,5	10,3	4,7	7,8	6,75	6,5	7,5
maximum	7,3				8,1	7,2	7,1	7,6
minimum	6,9				7,5	6,3	5,9	7,4
Standard Deviation	0,3				0,4	0,6	0,6	0,1
